# Internally Symmetrical Stwintrons and Related Canonical Introns in Hypoxylaceae Species

**DOI:** 10.3390/jof7090710

**Published:** 2021-08-29

**Authors:** Erzsébet Fekete, Fruzsina Pénzes, Norbert Ág, Claudio Scazzocchio, Michel Flipphi, Levente Karaffa

**Affiliations:** 1 Department of Biochemical Engineering, Faculty of Science, University of Debrecen, 4032 Debrecen, Hungary; penzesgirl03@gmail.com (F.P.); agnorbi@gmail.com (N.Á.); drir.michelflipphi@gmail.com (M.F.); levente.karaffa@science.unideb.hu (L.K.); 2 Juhász-Nagy Pál Doctoral School of Biology and Environmental Sciences, University of Debrecen, 4032 Debrecen, Hungary; 3 Section of Microbiology, Department of Infectious Diseases, Imperial College London, London SW7 2AZ, UK; c.scazzocchio@imperial.ac.uk; 4 Institute for Integrative Biology of the Cell (I2BC), Université Paris-Saclay, CEA and CNRS (UMR 9198), 91190 Gif-sur-Yvette, France

**Keywords:** spliceosomal intron, stwintron, (stw)intron proliferation, introner-like elements, symmetrical intronic sequences, fungal intron evolution, *Hypoxylon* sp. CO27-5

## Abstract

Spliceosomal introns are pervasive in eukaryotes. Intron gains and losses have occurred throughout evolution, but the origin of new introns is unclear. Stwintrons are complex intervening sequences where one of the sequence elements (5′-donor, lariat branch point element or 3′-acceptor) necessary for excision of a U2 intron (external intron) is itself interrupted by a second (internal) U2 intron. In Hypoxylaceae, a family of endophytic fungi, we uncovered scores of donor-disrupted stwintrons with striking sequence similarity among themselves and also with canonical introns. Intron–exon structure comparisons suggest that these stwintrons have proliferated within diverging taxa but also give rise to proliferating canonical introns in some genomes. The proliferated (stw)introns have integrated seamlessly at novel gene positions. The recently proliferated (stw)introns appear to originate from a conserved ancestral stwintron characterised by terminal inverted repeats (45–55 nucleotides), a highly symmetrical structure that may allow the formation of a double-stranded intron RNA molecule. No short tandem duplications flank the putatively inserted intervening sequences, which excludes a DNA transposition-based mechanism of proliferation. It is tempting to suggest that this highly symmetrical structure may have a role in intron proliferation by (an)other mechanism(s).

## 1. Introduction

Spliceosomal introns (U2 introns) are ubiquitous in eukaryotic nuclear transcriptomes. Their accurate excision requires the action of a highly specialised organelle, the U2 spliceosome, which catalyses two subsequent transesterification reactions crucially involving the terminal donor- and acceptor G’s at the 5′ and 3′ splice sites, respectively, and the internal lariat branch point A, releasing the intervening sequence in the form of a lariat intron RNA and fusing the bordering exons into a continuous open reading frame (recent reviews: [1,2,3]). Intron RNA is usually rapidly turned over, this process being initiated by the re-linearisation of lariat RNA by the action of the ubiquitous lariat debranching enzyme [4,5]. Nevertheless, some intron RNAs are more stable and able to perform crucial functions after being excised from pre-mRNAs (see reviews [6,7,8]).

**Figure 1:**
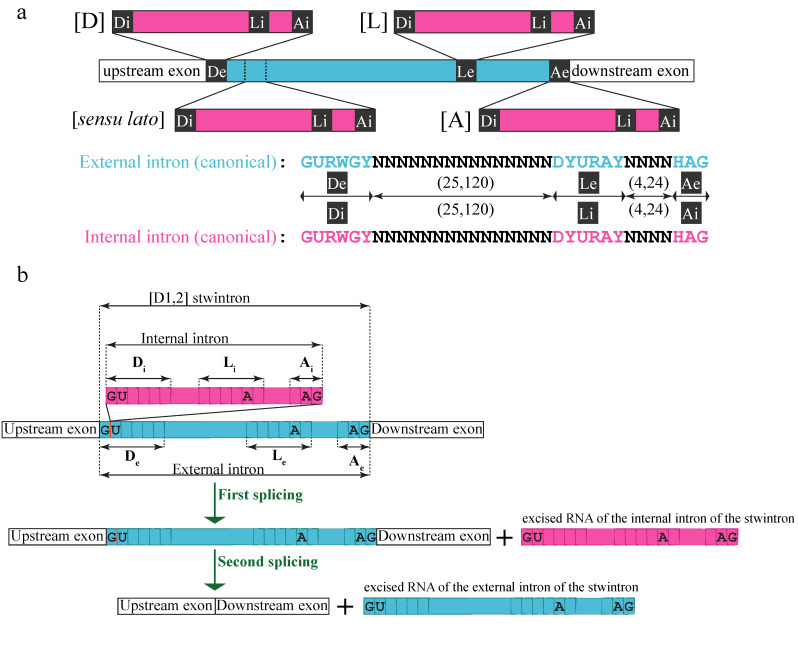
Structure and nomenclature of stwintrons and their typical stepwise excision. (**a**) A stwintron is a complex intervening sequence that consists of two nested canonical introns, which are excised by two subsequent standard U2 splicing reactions in “inside out” order [11]. There are four classes of stwintron, depending on which of the canonical sequence elements of the external U2 intron is disrupted by the internal U2 intron: (D), donor-disrupted; (L), BP element-disrupted; (A), acceptor-disrupted; (sensu lato), canonical intron within canonical intron, the former not included in (D), (L) or (A). (**b**) In a (D1,2) stwintron, an internal U2 intron (magenta) is nested within the 5′-donor element (D) of an external U2 intron (turquoise) between the first and the second nt of that donor element (5′-G_1_|U_2_). Removal of the internal intron from the pre-mRNA generates a functional donor for the external intron necessary for the excision of the latter; (D1,2) stwintrons in pre-mRNAs thus start with 5′-GGU (occasionally, 5′-GGC). The existence of the RNA intermediate of the consecutive U2 splicing reactions, which lacks the internal but still contains the external intron, can be experimentally verified. 5′-Donor, lariat branch point (BP) sequence element and 3′-acceptor of both constituent introns of the (D1,2) stwintron are annotated D_i_, L_i_ and A_i_, and D_e_, L_e_ and A_e_, respectively. Note that if the first exonic nt downstream of a (D1,2) is a G, the complex intervening sequence can be alternatively excised as an (A2,3) stwintron, in which the constituent introns swap order of excision [12,13]. This article deals exclusively with (D1,2) stwintrons, some of which are also (A2,3).

Intervening sequences may include more than one canonical intron unit [9,10]. We have described a class of nested U2 introns in ascomycete fungi, the stwintrons (**s**pliceosomal **tw**in **introns**) [11,12,13,14,15,16]. Stwintrons are removed from pre-mRNAs by two consecutive standard U2 splicing reactions (Figure 1a). Usually, either the 5′-donor, the sequence element around the lariat branch point (BP), or the 3′-acceptor of the external intron are interrupted by a second intron, the internal intron. However, we have also found functional stwintrons where the internal intron is integrated into the external intron close to, but not within, the latter’s six-nucleotide (nt) long consensus donor sequence (5′-GURWGY). In a (D1,2) stwintron (Figure 1b), the 5′ (internal) intron disrupts the 5′-donor sequence of the 3′ (external) intron between the first and the second nt (5′-G|URWGY). (NB. A (D1,2) stwintron thus starts with 5′-GGU). Excision of the internal intron results in a functional 5′ donor site for the external intron, enabling the latter’s excision. If the internal splice sites of the (D1,2) are masked or ignored by the spliceosome, the intervening sequence is not completely removed by one standard splicing reaction, and the first G of the (D1,2) stwintron becomes exonic, leading to a frameshift in the mRNA’s open reading frame.

The genesis of new spliceosomal introns—presently distributed across Pezizomycotina genomes at densities of ~3–4 introns per gene (e.g., [17])—remains a vexing mystery. Various mechanisms of intron acquisition have been proposed (reviews [18,19]). The availability of complete genome sequences of more than a thousand fungi provides unique opportunities to study intron genesis throughout a whole phylum as well as amongst closely allied species or variants. Fungal spliceosomal introns are generally small—often < 100 nt [20]. As a consequence, intron definition [21,22] is prevalent to pair the 5′-splice site and BP element that favour excision of the “smallest intron possible”. Canonical intronic sequence elements—5′-donor (six nt), the BP element (six nt), 3′-acceptor (three nt)—are well defined in model fungal genomes [20] and easily verifiable by comparative genomic analysis. These characteristics enabled the design of a simple motif search algorithm to predict potential stwintrons in fungal genomes in the absence of high-throughput transcription data [13,14,15]. By these means, we illustrated two mechanisms by which fungal (stw)introns are formed: by intronisation of bordering exonic sequences and by the formation of a new intron within an extant intron [14,15,16].

In this work, we uncover dozens of (D1,2) stwintrons and canonical U2 introns with a high degree of sequence similarity in the genomes of species of the *Hypoxylon* and *Daldinia* genera (Hypoxylaceae; Xylariales; Sordariomycetes; Pezizomycotina; Ascomycota). They appear to have integrated seamlessly into (previously) continuous exonic sequences at new intron positions. The proliferated (stw)introns appear to have been generated from a common (D1,2) template characterised by conserved terminal inverted repeats (45–55 nt), in parallel and independently in diverging taxa.

## 2. Materials and Methods

### 2.1. Data Mining, Gene Model, SRA Verification, and Functional Annotation of Genes with Sister (Stw)Introns in Species of Hypoxylaceae

Intervening sequences with high sequence similarity were collected from the genome of *Hypoxylon* sp. CO27-5 (GenBank Master Accession number MDCL00000000) upon BLASTN screening at the National Center for Biotechnology Information (NCBI, Bethesda, MD, USA), using the available online tools [23]. The (D1,2) stwintron we previously identified in the gene for a well-conserved mitochondrial carrier (named HCOc017A) (see Results Section 3.1) was the query sequence. The coding sequences (ATG–stop) of the genes harbouring sister (stw)introns were mined by TBLASTN screening of the Whole Genome Shotgun contigs (WGS) database at NCBI. The genome master accession numbers of the nine fungi in which sister (stw)introns were encountered are listed and referenced in Table 1.

Orthologue genes from other Hypoxylaceae species without sister stwintrons (*Hypoxylon* sp. CI-4A and *Annulohypoxylon stygium*, included in Table 1) were routinely collected. For a number of these genes, a deeper search for orthologues and paralogues in species of the Xylariales order was performed to track intron position conservation. We then manually predicted the intron–exon structure of each gene guided by comparative genomics. The sequence-derived information thus collected for each of the sister (stw)introns is shown in Supplementary Table S1 (four Excel sheets).

In *Hypoxylon* sp. CO27-5, *Hypoxylon* sp. EC38 and *Daldinia* sp. EC12, most of the gene model predictions could be corroborated by RNA sequence reads (relevant SRA accessions listed in Table 1). We identified perfectly matching SRA reads (size:100 nt) by BLASTN screening of the NCBI’s Sequence Read Archive for these three fungi, using 60 nt-long query sequences containing the predicted exon/exon fusion site at its centre. To detect the stwintron splicing intermediate (splinter)—the RNA species that lacks the predicted internal intron of the (D1,2) stwintron but still contains the latter’s external intron—we used query sequences (60 nt) containing in their centre, the predicted fusion site of the upstream exon and the external intron of the stwintron with its functional donor sequence intact. Because the splinter still contains the external intron of the stwintron and thus is not a splicing end product, its read count is generally (considerably) lower than that for the fully spliced RNA. SRAs that confirm sister (stw)introns and their predicted stwintron splicing intermediate in the three species mentioned above are listed in Supplementary Table S1. Contemporary automated annotation software does not recognise stwintrons, particularly not (D1,2) stwintrons that start with the noncanonical 5′-GGU (or occasionally, 5′-GGC) at the 5′-splice site. We did not use the results of automated annotation (“Models” or “mRNA” at the nr/nt database), nor did we use deduced protein databases to collect proteins. After translation of our predicted mRNAs, we functionally annotated most of the genes carrying sister (stw)introns with one or more protein domains categorised in the Protein Families (Pfam) database [31]. To construct the maximum likelihood tree of the mitochondrial carrier protein, we deduced the orthologue proteins for the 37 species of Xylariales order using our predicted gene models.

### 2.2. Other Informatics Methods

Multiple sequence Alignment with Fast Fourier Transform (MAFFT: version 7) was used for multiple sequence alignments [32]. To compare two or three intronic sequences across their entire span, Clustal Omega alignments were performed [33]. For the tree of the 37 mitochondrial carrier proteins, the alignment was trimmed with Block Mapping and Gathering with Entropy (BMGE: version 1.12) [34]. The maximum likelihood phylogenies of the *Hypoxylon* sp. CO27-5 sister (stw)introns and of the mitochondrial carrier protein were inferred using PhyML with Smart Model Selection (version 3.0) [35]. Branch support was estimated as detailed in the respective Figure legends. Trees were drawn with FigTree (version 1.4.3: http://tree.bio.ed.ac.uk/software/figtree, accessed on 4 October 2016) and further annotated with Adobe Illustrator (Adobe Inc., San Jose, CA, USA). RNAfold [36] was used to predict the secondary structure of single-stranded debranched intron RNAs with terminal inverted repeats (NB. Isolated base pairs were not avoided). RNAcofold [36,37] allows predicting the theoretical hybridisation energy of and the base-pairing pattern in double-stranded molecules of the same debranched (stw)intron RNA (NB. Isolated base pairs were not avoided). The sequence logo of the direct environment of the 5′- and 3′-(stw)intron/exon junctions in pre-mRNAs containing sister (stw)introns was created by Skyline [38] using default settings.

### 2.3. Hypoxylon sp. CO27-5, Growth Medium and Nucleic Acid Isolation

A monoculture of the endophytic fungus *Hypoxylon* sp. CO27-5 (Ascomycota; Pezizomycotina; Sordariomycetes; Xylariales; Hypoxylaceae; Hypoxylon; unclassified Hypoxylon) was maintained on Potato Dextrose Agar (PDA) (Neogen Culture Media, Lansing, MI, USA) [24]. For nucleic acid isolation, fungal biomass was generated in submerged cultures in 100-mL potato dextrose broth (HiMedia Laboratories GmbH, Einhausen, Germany) in 500-mL Erlenmeyer flasks, in a rotary shaker (Infors HT Multitron, Infors AG, Lonay, Switzerland) at 200 rotations per min for 24 h at 25 °C. Liquid cultures were inoculated with a dense suspension of mycelia, freshly scraped from the surface of one 7 days old PDA seed plate (96 mm diameter), in a sterile 1/10^4^ Tween-80 (VWR International LLC, Debrecen, Hungary) solution. Mycelial biomass was harvested by filtering the liquid culture over Miracloth (Millipore, Merck KGaA, Darmstadt, Germany) and thoroughly washed with sterile distilled water to remove oligosaccharide residue. Subsequently, the biomass was instantly deep frozen and ground to powder under liquid nitrogen. Genomic DNA and total RNA were isolated from the powdered biomass using the Macherey-Nagel NucleoSpin Plant II- and NucleoSpin RNA Plant kits, respectively (Macherey-Nagel GmbH & Co. KG, Düren, Germany).

### 2.4. Reverse Transcription Polymerase Chain Reaction (RT-PCR) and cDNA Sequencing

First strand cDNA was synthesised from total RNA template and Oligo(dT) as a primer using the RevertAid First Strand cDNA Synthesis Kit (Thermo Scientific, Thermo Fisher Scientific, Waltham, MA, USA). Using this single-stranded cDNA as the template, targeted PCR reactions were performed with gene-specific oligonucleotide primers (Integrated DNA Technologies, Leuven, Belgium) (Supplementary Table S2) and DreamTaq DNA Polymerase (Thermo Scientific, Thermo Fisher Scientific, Waltham, MA, USA), in a T100™ Thermal Cycler (Bio-Rad, Bio-Rad Hungary Ltd., Budapest, Hungary). Cycling conditions after initial denaturation at 95 °C for 2 min were: 35 cycles of 95 °C for 30 s, 60 °C for 1 min, and 72 °C for 0.5–1 min, followed by post-cyclic elongation at 72 °C for 5 min. Amplified DNAs were resolved in native agarose (SeaKem LE Agarose; Lonza Group Ltd., Basel, Switzerland) gels. As a control of efficacy, the performance of all designed gene-specific PCR primers was verified with genomic DNA template.

To confirm the predicted stwintron splicing intermediate (splinter), we designed primer pairs that do not amplify first strand cDNA of fully spliced mRNA. For (D1,2) stwintrons, the gene-specific reverse primer hybridises entirely or partly (i.e., at its 3′ end) with a sequence within the external intron. This strategy usually yields two PCR fragments of which the smaller one corresponds to the stwintron splicing intermediate. All experiments were done in duplicate, starting with biomass from two independent liquid cultures.

Double-stranded cDNA was gel-purified (NucleoSpin Gel & PCR Clean-up, Macherey-Nagel GmbH & Co. KG, Düren, Germany), then cloned in pGEM-T Easy (pGEM-T Easy Vector System I, Promega Corporation, Madison, WI, USA). Plasmid DNA was isolated using the NucleoSpin Plasmid EasyPure kit (Macherey-Nagel GmbH & Co. KG, Düren, Germany). Three independent clones were sequenced over both strands using universal primers hybridising to the vector (Eurofins Genomics, Ebersberg, Germany) or gene-specific oligonucleotide primers where appropriate. cDNA and splinter sequences from *Hypoxylon* sp. CO27-5 were deposited at GenBank under accession numbers MW490712–MW490722, MW498245–MW498262 and MW530466–MW530509.

## 3. Results

### 3.1. Identification of a New (D1,2) Stwintron in the Gene for a Mitochondrial Carrier Protein in Two Allied Hypoxylon

While investigating the intron–exon structure of a gene for a well-conserved, 339 amino acid-long mitochondrial carrier in *Aspergillus nidulans* (*A. nidulans* GenBank AACD01000130, CDS coordinates start-stop: 208790-210069, unpublished work) with comparative genomics, we noticed an archetypal (D1,2) stwintron in the orthologue gene in the *Hypoxylon* sp. CO27-5 and EC38 genome sequences, absent from all other fungi assessed. When the CO27-5 genome was back screened with the stwintron’s sequence (BLASTN), dozens of full length and partial hits were uncovered, suggesting that this new (D1,2) stwintron is part of a set of structurally related intervening sequences.

### 3.2. Abundance of Highly Similar (D1,2) Stwintrons in the Genome and Transcriptome of Hypoxylon sp. CO27-5

**Figure 2:**
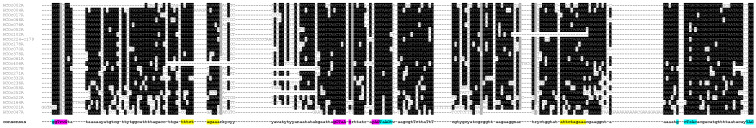
Alignment of 23 (D1,2) sister stwintrons from *Hypoxylon* sp. CO27-5. 23 (D1,2) stwintrons with high global sequence similarity were collected by BLASTN screening of the genome sequences of *Hypoxylon* sp. CO27-5 (GenBank MDCL00000000: 580 contigs) with the (D1,2) stwintron in a conserved mitochondrial carrier gene (stwintron HCOc017A) as the query. The sequence of stwintron HCOc224-179 and the gene harbouring it (*duf636*) was determined experimentally (GenBank Accession MW477887). The full-length intronic sequences were aligned by Multiple Sequence Alignment (MAFFT) using E-INS-i iterative refinement and the 20PAM scoring matrix without sequence trimming. The consensus line was annotated with IUPAC symbols for combinations of bases occurring on each of the 276 positions, except for the apparent insertions that had occurred in only one or two sequences. 5′-Donor-, (predicted) BP- and 3′ acceptor sequences are highlighted in colour—magenta for the internal intron and turquoise for the external intron. The two copies of a 10-nt palindrome (5′-WTTCTAGAAA) are highlighted in yellow background.

In all, we identified with a simple BLASTN search 23 (D1,2) stwintrons in *Hypoxylon* sp. CO27-5, which are highly similar in sequence over the complete width. An alignment is shown in Figure 2. The BLASTN query stwintron is named HCOc017A, where HCO refers to the fungus *Hypoxylon* sp. strain CO27-5, c017 refers to the sequence contig in which it occurs (i.e., GenBank MDCL01000017), and the terminal capital letter (here, A) identifies the individual stwintron(s) in that contig.

The global similarity of the 22 stwintron sequences ranged between 58 and 90% of identity with the query, HCOc017A. We have termed these 23 stwintrons **sister stwintrons** to reflect their similarity and the likelihood of common ancestry. They are located in 22 different contigs, scattered throughout the genome (Supplementary Table S1: sheet CO27-5). One sister stwintron (HCOc224-179) is split over two non-overlapping CO27-5 sequence contigs due to an assembly artefact: we have determined its full-length sequence (GenBank accession number MW477887). Repetitive sequence elements that correspond to functional intronic sequences are called “introners” [39] and have been reported in a number of organisms in different eukaryotic kingdoms, including in fungal taxa [39,40,41,42,43,44,45,46,47,48,49,50], but the proliferation of complex intervening sequences, e.g., stwintrons, has never been described before.

In addition, we identified 13 additional (D1,2) stwintrons with a high similarity region restricted to a section of the complex intervening sequence. (Supplementary Table S1: sheet CO27-5). In HCOc103A, the external intron is very similar to the external introns of “full-length” sister stwintrons, but its internal intron is unique in the *Hypoxylon* sp. CO27-5 and *Hypoxylon* sp. EC38 genomes. Seven of these 13 **sheared sister stwintrons** conserve the central section, where the two consecutive canonical introns overlap by the terminal G (AGT: typical for (D1,2)). All 36 (D1,2) stwintrons in *Hypoxylon* sp. CO27-5 identified with BLASTN screenings are listed in Supplementary Text Document S1. For each of the sheared sister stwintrons, we highlighted the patch homologous to HCOc017A by underlining and the yellow background in the Supplementary Text Document S1. All but two of these 36 sister stwintrons are also present in the genome of the closely related strain *Hypoxylon* sp. EC38.

### 3.3. Experimental Identification of Sister Stwintrons

We have verified the typical two-step splicing of the predicted stwintrons and their splicing intermediate using the available RNA SRAs (Sequence Read Archive) at NCBI. In parallel, we have sought to experimentally confirm both the stwintron splicing intermediates and the complete (D1,2)s by targeted RT-PCR and cDNA sequencing using specific oligonucleotide primers (detailed in the Materials and Methods section). See Supplementary Table S1 for SRA evidence and GenBank accession numbers. By these complementary means, we could confirm the two-step excision for 31 out of 36 predicted stwintrons. The transcripts of the genes in which four of the remaining predicted (D1,2) stwintrons are located could not be amplified by RT-PCR, implying that these genes are not expressed under the growth conditions used to generate template RNA (see Materials and Methods section).

### 3.4. Identification of Canonical U2 Introns with High Sequence Similarity to the above (D1,2) Sister Stwintrons in Hypoxylon sp. CO27-5

**Figure 3:**
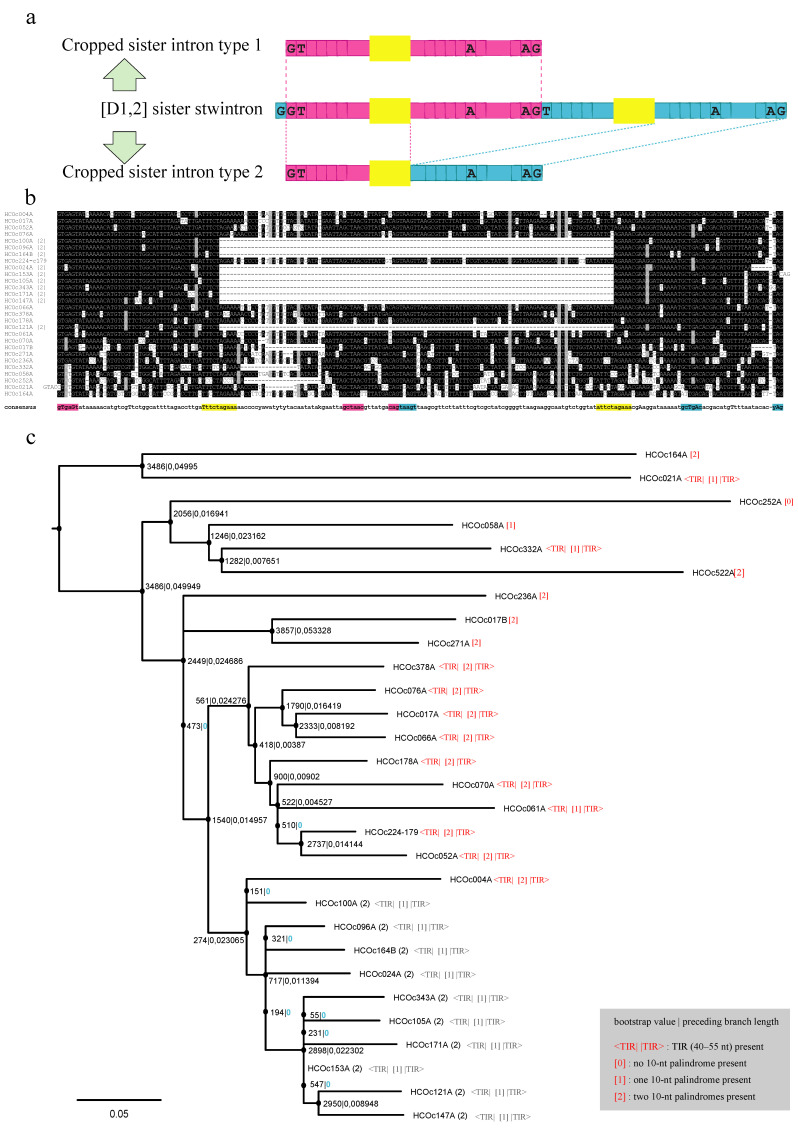
Two types of cropped sister introns derive from different sections of a sister stwintron template. (**a**) Type-1 cropped sister introns (magenta) correspond to the internal introns of sister stwintrons (top), while type-2 cropped sister introns correspond to the 5′ half of the internal intron (magenta) fused to the 3′ half of the external intron (turquoise) (bottom). The yellow boxes represent the two copies of the 10-nt palindrome 5′ WTTCTAGAAA extant in sister stwintrons. Cropped sister introns feature only one such palindrome. (**b**) Alignment of 10 type-2 cropped sister introns with the 18 most similar (D1,2) sister stwintrons. Sequences were aligned and the consensus intron sequence elements (5′-donor; BP-sequnce, 3′-acceptor, 10-nt palindrome) of the constituent canonivcal introns are colour-marked as described in Figure 2. Eight sister stwintron sequences were manually trimmed by removing single nt or longer insertions such that the number of informative nt was 206. None of the type-2 cropped sister intron sequences were manually trimmed. (**c**) Maximum likelihood phylogeny of 19 paralogue sister stwintrons and 10 type-2 cropped sister introns in *Hypoxylon* sp. CO27-5. The intervening sequences were aligned by MAFFT using the E-INS-i iterative refinement module and the 20PAM scorings matrix. Terminal duplications in HCOc121A and HCOc153A (2) were removed. Type-2 cropped sister introns consists of only the overlapping TIRs. A maximum-likelihood tree was inferred from the untrimmed MAFFT alignment (249 informative nt) by SMS-PhyML running 5000 iterations of Felsenstein’s bootstrap [51] to estimate branch support. The scale bar represents 0.05 substitutions per site. At the nodes, bootstrap figures are given along with the length of the preceding branch (separated by a vertical bar): branches of “zero” length are highlighted in green. There are no substitution models specially developed for phylogenies of intervening sequences: GTR + G + I was automatically selected as the substitution model. Next to each sister stwintron name, we indicated in red lettering whether terminal inverted repeats (TIRs) and palindrome copies are present (see Section 3.5 for the criteria). We used grey letters to indicate the same information for the 10 type-2 cropped sister introns near the bottom of the tree. All symmetrical elements are considered to be present in 9 of the 10 least divergent (viz. shortest branch lengths) sister stwintrons clustered near the bottom of the tree. The corresponding estimations for the presence of the TIRs and the palindromes for the four stwintrons absent from the phylogeny are HCOc121A: <TIR| (1) |TIR>; HCOc406A: (1); HCOc002A: (1); HCOc047A: (0).

Furthermore, we found 12 canonical introns that show a high similarity to a section of the complete sister stwintron sequence (Figure 3a; intron sequences listed in supplementary Text Document S2). In two instances to be called **type-1 cropped sister introns** (HCOc047B and HCOc229A; Supplementary Figure S1), the similarity extends to the whole internal intron; that is, they appear to result from the deletion of the external intron [13,14]. In 10 other instances, **type-2 cropped sister introns** consist of a fusion of 5′ sequences of the internal intron with 3’ sequences of the external intron (see alignment in Figure 3b), and, therefore, they must result from deletion of the central region in the corresponding genomic DNA. The 10 type-2 cropped sister introns constitute a more homogeneous group of sequences than the 23 sister stwintrons, suggesting that they have formed more recently than most of the latter and originate from a stwintron ancestor.

This is coherent with the evolutionary relations amongst 19 sister stwintrons and the 10 type-2 cropped sister introns inferred from a maximum likelihood phylogeny of these paralogue intervening sequences in *Hypoxylon* sp. CO27-5 (Figure 3c). Indeed, three of the 10 type-2 cropped sister introns (HCOc105A; HCOc343A; HCOc171A) are unique to strain CO27-5 (i.e., they do not occur in EC38). We confirmed 11 of the 12 cropped sister introns, either with extant SRA data (at NCBI) or by RT-PCR (Supplementary Table S1: sheet CO27-5). We could not amplify cDNA from the transcript of the gene carrying type-2 cropped sister intron HCOc096A. Moreover, *Hypoxylon* sp. EC38 also harbours three unique type-2 cropped sister introns, absent from CO27-5 (Supplementary Table S1: sheet EC38).

The loss of the central stwintron sequence when a type-2 cropped sister intron originates from a sister stwintron appears to occur by recombination involving a 10-nt palindrome, 5′-WTTCTAGAAA, a small direct repeat present within many of the sister stwintrons (Supplementary Table S1). The 5′ copy (in most cases, 5′-W = T) is roughly located in the centre of some of the (D1,2)s’ internal intron and the 3′ copy (always imperfect: 5′-W = A in all but one case) roughly in the centre of the external intron, while the type-2 cropped sister introns retain in all but one case of one centrally located, perfect palindrome, 5′-TTTCTAGAAA.

The underlying mechanism to generate a canonical sister intron from an extant sister stwintron is suggested by the intervening sequences at the fifth intron position in the orthologous *Hypoxylon* sp. CO27-5 and EC38 genes for an integral membrane protein of 413 amino acids (Supplementary Table S1, sheets CO27-5 and EC38). In strain EC38, the phase-two type-2 cropped sister intron HECc034A (93 nt) is shifted by one nt in position as compared to the phase-one sister stwintron HCOc066A (207 nt) extant in strain CO27-5. The alignment of the two “orthologous” intervening sequences (Supplementary Figure S2) shows that the central part of the stwintron—including its internal splice sites—was absent from the EC38 type-2 cropped sister intron, as well as one copy of the 10-nt palindrome.

Deletion of intronic sequences could occur if a double-stranded DNA break (DSB) located between the two 10-nt palindrome copies in the stwintron was repaired by microhomology-mediated end-joining (MMEJ) (reviews [52,53]). The observed pattern of deletion is typical for DSB repair by MMEJ, where the 10-nt palindrome provides the necessary microhomologies on either side of the break (Supplementary Figure S3). A key enzyme in MMEJ in metazoans is Polymerase θ (POLQ). Many taxa of Pezizomycotina include a gene encoding a superfamily-II DNA/RNA helicase, homologous to the N-terminal helicase domain in Polymerase θ [54,55,56] but without a polymerase domain. In *Hypoxylon* sp. CO27-5, putative POLQ-like helicase mRNA and protein are extant (named HelQ; see GenBank accession numbers MW530496 and MW530509). In Hypoxylaceae, this clearly identifiable helicase gene includes a (D1,2) stwintron (albeit not a sister stwintron).

The transition of a (D1,2) stwintron to a canonical intron implies that the 5′-donor G_1_ of the external intron of the stwintron becomes exonic, and secondary mutations being required to restore the reading frame of the mature mRNA. In this case, (HECc034A), secondary mutations adjacent to either intron–exon junction resulted in a transversion of the exonised G_1_ into a T with a concomitant shift in intron position, by which the Thr codon (A|CC) split by the phase-one stwintron (CO27-5) is changed to an Ile codon (AT|C) split by the phase-two canonical intron (EC38), with the loss of one of the exonic Cs directly downstream the ancestral stwintron. Other *Hypoxylon* species do not carry an intron at or near that position, but almost all specify a Thr codon there, suggesting that the EC38 cropped sister intron is formed from a sister stwintron orthologous to the one in CO27-5, and not vice versa.

Six type-2 cropped sister introns (HCOc105A; HCOc121A; HCOc147A; HCOc153A; HCOc171A; HCOc343A) constitute a separate group (Figure 3b,c; Supplementary Text Document S2). Near their 3′, they have an additional G directly upstream the acceptor (except in HCOc153A, where a CA duplication is apparent 3′of that additional G)—no sister stwintron have this extra G. Furthermore, 7 nt upstream of the typical additional G, these six type-2 cropped sister introns have a T, where the other four, as well as all sister stwintrons, have an A. Finally, the four type-2 cropped sister introns not belonging to this group all have a TAG acceptor, while five of the group of six feature a CAG acceptor (HCOc343A ends with the less-canonical 5′-GAG: Supplementary Table S1). The existence of this clade of six strongly suggests that type-2 cropped sister introns proliferate independently from sister stwintrons. Note that three of the six, HCOc105A, HCOc171A, and HCOc343A, are unique to CO27-5.

### 3.5. Symmetrical Characteristics of Type-2 Cropped Sister Introns and Sister Stwintrons

**Figure 4:**
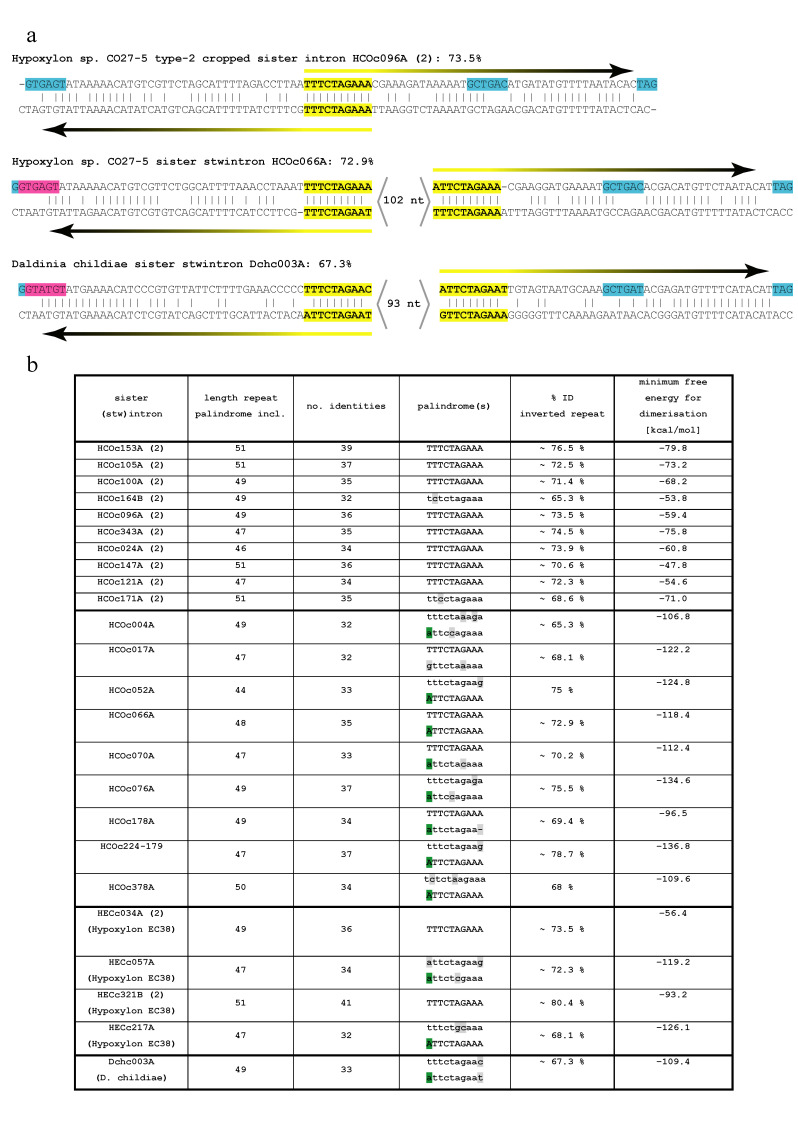
Terminal symmetry in type-2 cropped sister introns and sister stwintrons. (**a**) Examples of type-2 cropped sister introns and sister stwintrons aligned with their own reverse complement sequence to show considerable terminal symmetry. Clustal-Omega was run with default settings. The 5′-donor, BP and 3′-acceptor elements of the internal (magenta) and external (turquoise) introns are highlighted on the coding strand. Terminal repeats (44–55 nt) are indicated by the horizontal arrows. The 10-nt palindrome (5′-WTTCTAGAAA) is highlighted in yellow. For the sister stwintrons, the space between the two copies of the 10-nt palindrome—including the internal splice sites—is indicated: <nt>. The percentages of nt identity of the terminal repeats are given for each of the three sister (stw)introns shown as examples. (**b**) The percentage of identity of the terminal inverted repeat in all 10 type-2 sister introns and the 9 least divergent sister stwintrons. Corresponding data for four sister (stw)introns in *Hypoxylon* sp. EC38 are included, as well as for one *Daldinia childiae* sister stwintron, Dchc003A (see Section 3.7). The sequences of the 10-nt palindromes are given in capital letters where perfect: The 5′ A in the 3′ palindrome copy is highlighted in green and present in all but one of the 12 sister stwintrons analysed. Other divergencies are highlighted in grey. The theoretical minimum free energy of double-stranded sister (stw)intron RNA molecules (right column) was predicted by RNAcofold.

The 10 type-2 cropped sister introns not only resemble one another but also feature extensive similarity with their own reverse complement sequences. Figure 4a shows an alignment of HCOc096A (93 nt) and its reverse complement, revealing the symmetry of its terminal sequences, as an imperfect inverted repeat (49 nt) overlapping at and by the central 10-nt palindrome 5′-TTTCTAGAAA, with ~73.5% sequence identity. A total of 9 of the 23 sister stwintrons (Figure 4b) also feature a clear terminal inverted repeat (TIR) of considerable length (45–55 nt), revealed by a straightforward alignment of each stwintron with its own reverse complement sequences.

The TIRs in one sister stwintron, HCOc066A (207 nt), 48-nt long with ~72.9% sequence identity and separated by 102 nt, are also shown in Figure 4a. The phylogeny of 19 sister stwintrons and 10 type-2 cropped sister introns (Figure 3c) includes, after each sister stwintron name, an estimation of the presence of the TIRs revealed by the alignment with their respective reverse complement sequences, as well as of the palindrome 5′-WTTCTAGAAA within the TIR, considered present when its sequence is conserved for >7 of its 10 nt. A total of 9 of the 10 least divergent (i.e., probably the most recently evolved) CO27-5 sister stwintrons have clear TIRs and both copies of the 10-nt palindrome, while in HCOc061A (the tenth), one of the palindromes is somewhat degenerated. For most of the nine earlier divergent (D1,2)s in the phylogeny (Figure 3c), clear TIRs could not be identified by simple alignment with their reverse complement sequences, while often, one or both 10-nt palindromes are degenerated or absent. The 33 highly similar sister (stw)intron RNAs in *Hypoxylon* sp. CO27-5 could not be assigned to any known RNA family in the Rfam database (version 14.3: 3446 families) [57], which contains highly structured, self-splicing RNAs as well as intron-born, small nucleolar RNAs (aka, snoRNAs). They also do not bear primary sequence similarity to any described family of fungal introner-like sequences in Dothideomycete species of the Capnodiales order [42,44,45,46,48,50].

The expression data (SRAs/RT-PCR) strongly suggest that terminal symmetry—including the intronic 10-nt palindrome (5′-WTTCTAGAAA)—is not necessary for (stw)intron removal by the U2 spliceosome (see Supplementary Table S1). A remarkable feature of the TIRs in the *Hypoxylon* sister (stw)introns is that frequently, the three invariant nucleotides involved in the two transesterification reactions of U2 excision—the 5′-terminal donor G, the lariat branch point A, and the 3′-terminal acceptor G—are not base pairing in the predicted hairpin structure in single-stranded (ss) RNA (not shown). Despite the symmetry, a genuine (stw)intron sequence is only present on the coding DNA strand.

### 3.6. Sister (Stw)Intron Integration Sites Are Apparently Not Biased

We further investigated the exons bordering each of the 48 identified intervening sequences in *Hypoxylon* sp. CO27-5, and compared them to orthologue genes in other Hypoxylaceae and Xylariaceae species (Supplementary Table S1). Two sister stwintrons (HCOc076A and HCOc102A) and one type-1 cropped sister intron (HCOc047B) are located in sequences that seem unique to *Hypoxylon* sp. CO27-5 and EC38. One sheared sister stwintron (HCOc046A) is located in the 5′ untranslated region of the host gene, 29 nt upstream of the predicted start codon. Sister stwintron HCOc021A occurs in a recently pseudogenised gene (frameshift mutations) for a short-chain dehydrogenase/reductase. A total of 42 of the 43 other intervening sequences are inserted seamlessly in the coding regions, i.e., without loss or gain of exonic sequences. Type-2 cropped sister intron HCOc121A in CO27-5/EC38 (the 43rd) is replacing three nt locally present in other *Hypoxylon* species which are intron-less at this position.

**Figure 5:**
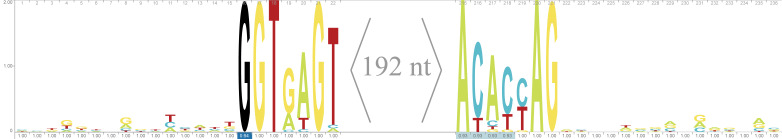
Sequence logo of the (stw)intron–exon junctions of 28 sister (stw)introns in *Hypoxylon* sp. CO27-5. The logo visualises the extent of nucleotide conservation in a multiple sequence alignment and highlights conservation patterns adjacent to the (stw)intron–exon junctions of sister (stw)introns. The 28 sister (stw)intron sequences in the trimmed alignment shown in Figure 3b were extended to include their respective bordering exonic sequences (15 nt on each side). For convenience, the intron–exon junctions were aligned precisely by further manually trimming of the 5′ terminus of HCOc021A and the 3′ terminus of HCOc153A (2), while the additional G (near 3′) unique to six of the type-2 cropped sister introns (see Section 3.4) was also removed. However, all exonic sequences included in the input alignment of the logo are authentic. Observed counts were used in processing. The relative sizes of the four nts indicate their frequency at each individual position. The height of the nt stack equals the information content at each position. (D1,2) stwintrons start with GGT (GGU): the black G (position 16) in the logo is thus intronic and corresponds to the 5′-donor G_1_ of the external intron of the stwintron (occupancy: ~0.64). For clarity, only the sections of the logo in the direct proximity of the (stw)intron–exon junctions are shown.

Figure 5 shows a compact graphical representation of conservation patterns near the intron–exon junctions for 18 sister stwintrons and 10 type-2 cropped sister introns.

In contrast to the high level of similarity within these intervening sequences, no similarity or pattern was obvious in the exonic sequences (15 nt) directly bordering them. Moreover, no canonical splice sites (donor or acceptor) are duplicated at the site of integration. The sister (stw)intron insertion sites were not biased toward any one phase, nor could we discern a positional bias for integration within the pre-extant intron–exon structures of the CO27-5 genes carrying the 48 intervening sequences under study (Supplementary Table S1: sheet CO27-5). A total of 7 of the 33 sister (stw)introns (~20%) occur in genes without other intervening sequences. An outstanding example is the gene (HCOc153A) for an oxidoreductase from the nitrilotriacetate monooxygenase family (447 amino acids), for which the protein product is >84%, identical to orthologues in Rhizobiales (alphaproteobacteria). On the other hand, a gene coding for a protein of 676 amino acids with a SIR2-like domain harbours a sister stwintron (HCOc164A) at the second position and a type-2 cropped sister intron (HCOc164B) at the third intron position. This gene has thus been subjected twice to different intron proliferation events.

### 3.7. Recent Emergence of the Structurally Related Sister Stwintrons and Derived Type-2 Cropped Sister Introns

A total of 27 of the sister stwintrons and type-2 cropped sister introns in CO27-5 are present at new intron positions when comparing the gene models of the orthologue genes in the order of the Xylariales (in 3 other cases, there is no orthologue gene; see Section 3.6). Apart from the sister (stw)introns that are unique to *Hypoxylon* sp. CO27-5, 20 sister stwintrons and most of the type-2 cropped sister introns identified occur in both *Hypoxylon* sp. CO27-5 and EC38 but not in *Hypoxylon* sp. E7406B or *H. pulicicidum* (nor in more remotely related Xylariales species). In the HCOc103A stwintron, only the external intron is similar to the external introns of “full-length” sister stwintrons (Supplementary Text Document S1 and Supplementary Table S1). This sheared sister stwintron also occurs uniquely in *Hypoxylon* sp. CO27-5/EC38. On the other hand, 9 of the 12 other sheared sister stwintrons, with high similarity restricted to a section of the intervening sequence, are present in all four above mentioned *Hypoxylon* genomes. In the three remaining cases—stwintrons HCOc046A, HCOc091B and HCOc304A—the orthologue gene was absent from E7406B/*H. pulicicidum*. This pattern of occurrence suggests that the 33 sister stwintrons and type-2 cropped sister introns of high sequence similarity have formed after the divergence of *Hypoxylon* sp. CO27-5/EC38 and *Hypoxylon* sp. E7406B/*H. pulicicidum*, while the sheared sister stwintrons are older. Although we cannot rule out that the three cropped sister introns unique to CO27-5 have been lost again from EC38 after their inception, it is more likely that they were generated after the separation of these closely related organisms. This assessment is supported by the presence of a new sister stwintron and a new type-2 sister intron in EC38 in two genes that also carry a position-conserved sister stwintron shared by CO27-5 and EC38 (Supplementary Table S1: sheet EC38). The EC38 gene encoding a putative 770 amino-acid-long small oligopeptide transporter harbours the HECc321A stwintron corresponding to CO27-5 HCOc271A, as well as a new cropped sister intron, HECc321B (2), 582 nt downstream. Similarly, the EC38 gene encoding a putative 593 amino acids-long multicopper oxidase harbours, as a new sister stwintron, (HECc217A) 71 nt upstream the sister stwintron shared with CO27-5, HECc217B, and HCOc003A, respectively (Supplementary Figure S4).

We subsequently assessed the presence of sister stwintrons similar to the one in the CO27-5/EC38 mitochondrial carrier gene (HCOc017A) in other species of Hypoxylaceae and in Xylariaceae. We found (D1,2) stwintrons highly similar to CO27-5/EC38 sister stwintrons in the genomes for *Hypoxylon* sp. E7406B and *H. pulicicidum* (for sequences: Supplementary Text Document S3), but perhaps more remarkably, also in some more remotely related species, *Hypoxylon rubiginosum* and the four sequenced *Daldinia* (*D. eschscholzii*; *Daldinia* sp. EC12; *D. childiae*; *D. concentrica*) (Supplementary Text Document S4). The phylogenetic tree in Supplementary Figure S5 (aLRT node statistics: [58]) shows the relationships between Hypoxylaceae taxa with and without sister (stw)introns for the well-conserved mitochondrial carrier encoded in *Hypoxylon* sp. CO27-5 by the gene harbouring sister stwintron HCOc017A. We found up to 18 putative sister stwintrons in the *D. childiae* genome sequences and five in *H. rubiginosum*. Figure 4 and Supplementary Figure S6 include comparative analyses of the typical TIRs in one of the sister stwintrons found in *D. childiae*, Dchc003A. For *Daldinia* sp. EC12, we have collected RNA SRA evidence for the existence of five of the six predicted sister stwintrons (Supplementary Table S1: sheet Daldinia EC12). Remarkably, no cropped sister introns could be predicted in *Daldinia* or in *H. rubiginosum*.

In *Hypoxylon* sp. E7406B and *H. pulicicidum*, we found nine sister stwintrons, eight of which were present in both these fungi (Supplementary Table S1: sheet E7406B). One sister stwintron was unique to E7406B (HE7c137A), while an additional sister stwintron was also found in a sequence unique to *H. pulicicidum* (Hpuc023A). Furthermore, one type-2 cropped sister stwintron was encountered in both E7406B (HE7c057A) and *H. pulicicidum* (Hpuc021A). This cropped sister intron is present in the very same gene that carries the CO27-5 sister stwintron HCOc378A, encoding a putative transporter of the AzgA family. Intriguingly, the two taxon-specific but primary sequence-related intervening sequences are integrated at opposite ends of the coding region and thus must have been generated independently. In fact, none of the 11 sister (stw)introns in E7406B and/or *H. pulicicidum* were present in orthologue genes in *Hypoxylon* sp. CO27-5/EC38. This strongly suggests that they have emerged after the separation from the CO27-5/EC38 taxon, albeit from essentially the same template sequence.

## 4. Discussion

We have found dozens of introns and (D1,2) stwintrons in fungal species of the *Hypoxylon* and *Daldinia* sister genera exhibiting high global primary sequence similarity, suggesting that they have originated from a common ancestral intervening sequence. The canonical type-2 cropped sister introns in *Hypoxylon* sp. CO27-5 show an extraordinary symmetry, with ~45–55 nt long TIRs overlapping at a central palindromic sequence (5′-TTTCTAGAAA). In the least divergent (conceivably the more recently emerged) sister stwintrons (Figure 3c and Figure 4b), TIRs of essentially identical length are present, albeit separated by some 100 nt between the two copies of the 10-nt palindrome, absent from the abbreviated type-2 cropped sister introns. Nevertheless, both the stwintrons and the canonical derivates must have been able to propagate. These intervening sequences are predicted to form hairpin- or stem-loop secondary structures (not shown), but the symmetry is such that we predict that two molecules of spliced out and debranched sister intron RNA may also base pair to form a double-stranded (ds) RNA species (see below). Our results suggest that neither the TIRs nor the 10-nt palindrome(s) are necessary for excision by the U2 spliceosome (Figure 3c: annotation in red lettering; Supplementary Table S1), but their conservation in the least divergent sisters stwintrons suggests that the TIRs may be crucial for the observed (stw)intron proliferation.

Sister (stw)introns (with two exceptions) occur at new intron positions within the coding region of the resident genes, seamlessly integrated (one exception, see Section 3.6) in exonic sequences which are continuous in other Hypoxylaceae species. There are three EC38 genes with two sister (stw)introns, suggesting that insertions are not randomly distributed across the genome. Half of the genes interrupted by sister (stw)introns (17/33) have paralogues without intervening sequences at the corresponding position elsewhere in the genome, often with a similar underlying intron–exon structure. Furthermore, seven sister (stw)introns (7/33) occur in genes without any other intervening sequence. There are no conserved patterns in the exons bordering sister (stw)introns at either the nucleotide- or the codon level (see Figure 5 and Supplementary Table S1). Intron phase bias was not observed, nor was there a preference for (de novo) intron generation towards the 5′ terminus of primary transcripts, all suggesting random integration events at the genomic DNA level. However, all integrated symmetrical sequences are only functional as intervening sequence(s) on the coding strand, hinting towards a link with transcription.

Many of our observations for *Hypoxylon* sister (stw)introns are very similar to those described for the diverse families of introner-like elements in taxa of Capnodiales [44,45,46], which are canonical U2 introns, particularly, the seamless integration in seemingly random exonic sequences, the presence of related repetitive intron sequences propagating in diverging taxa and the symmetrical structure of fungal introner-like elements. Van der Burgt and co-workers [45] refer to “predicted stable secondary structures” rather than symmetry, while Torriani and co-workers [44] discern “inverted repeats”. Crucially, this structural characteristic distinguishes fungal introner-like elements from the original introners in the green algae *Micromonas* that “lack known transposable element characteristics” (for the definition of introners, see [39]). In *Hypoxylon*, we observe degeneracy of the internal symmetry in the more divergent sister stwintrons (Figure 2 and Figure 3), supporting the hypothesis that at the species level, many regular spliceosomal introns could, in fact, be introner-like elements which sequences have degenerated beyond recognition [45,46].

We established that the introner concept of intron propagation extends to complex intervening sequences, albeit we found 23 sister stwintrons and not hundreds or even thousands of copies such as for the fungal intron-like elements originally defined by Collemare et al. [46]. In contrast to van der Burgt and co-workers [45], we could not discern a preference of integration at AG/GY sites nor in codon phases in *Hypoxylon*. Despite exhibiting a low duplication frequency, we found functional canonical introns we named type-1 cropped sister introns that are repetitive in sequence but not symmetrical, formed from a sister stwintron by one standard intron loss event similar to those previously described by us [11,14]. In either case, the external intron of the (D1,2) was lost, but the loss of the internal intron would be equally attainable.

**Figure 6:**
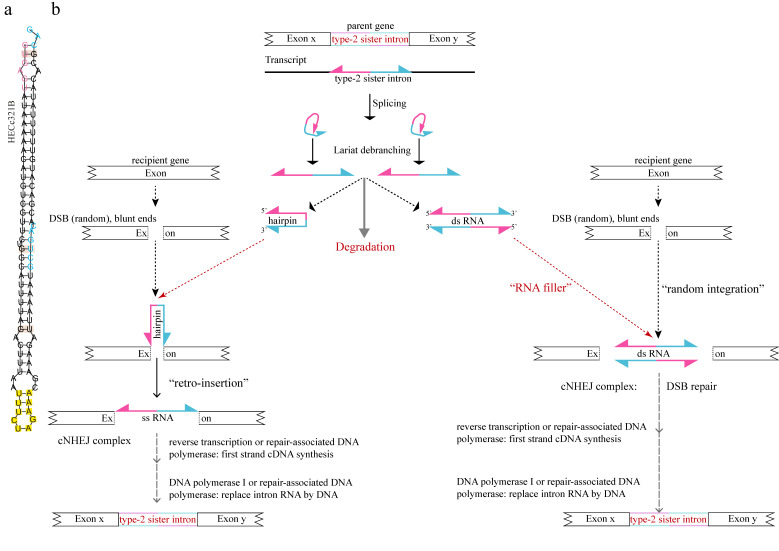
Proposed mechanisms to account for the proliferation of a highly symmetrical type-2 cropped sister intron during DSB repair. (**a**) Hairpin structure of HECc321B single-stranded intron RNA as predicted by RNAfold. The three noncanonical GU base pairings (salmon pink) and the 10-nt palindrome 5′-TTTCTAGAAA (yellow) are marked by differently coloured backgrounds. The 5′-donor, 3′-acceptor and BP elements are highlighted magenta or turquoise. (**b**) Two proposed duplication mechanisms of introns of highly internal symmetry. A schematised type-2 cropped sister intron RNA is shown in magenta (5′ half) and turquoise (3′ half) colours. Central at the top of the panel, the scheme shows the possible secondary structure formation, while the schemes to the right and to the left, respectively, show two possible mechanisms of intron integration at novel genomic sites. A self-folded, single-stranded intron RNA is formed (hairpin), or, alternatively, a ds-RNA molecule is formed by annealing debranched molecules of single-stranded RNA. We assume that most unfolded intron RNAs will be rapidly degraded. A DSB would occur at the onset of sister intron duplication at a new genomic locus. To the left of figure panel, the single-stranded intron RNA is inserted in its new position between the broken ds-DNA ends tethered together by the cNHEJ complex. To the right, the double-stranded molecule of the internally symmetrical intron RNA would be inserted in the DSB in a process resembling random integration of extrachromosomal DNA. (D1,2) sister stwintron duplication essentially follows the same steps, driven by its TIRs present in a rare alternative splicing product consistent with the whole stwintron sequence with the exception of the 5′-G_1_. (see Discussion Section 4).

However, the most relevant novelty comes from the impromptu formation of symmetrical canonical introns we called type-2 cropped sister introns by the deletion of the central part of a sister stwintron in the *Hypoxylon* sp. CO27-5/EC38 taxon. These abbreviated intervening sequences, half the size of a sister stwintron, are clearly capable of duplication (Figure 3 and Figure 4). Their existence and propagation imply that intron-duplicating ability primarily correlates with their highly symmetrical structure and not with their size. The cropping towards the termini resulted in introns that only consist of terminal-inverted repeats, increasing the level of symmetry. Figure 6a shows the predicted folding of the most symmetrical type-2 cropped sister intron RNA we have encountered, HECc321B. This implies that the ~45–55 nt long TIRs in the 10 type-2 cropped sister introns would be both necessary and sufficient for the generation of new sister introns. This is corroborated by the increasing degree of degeneracy of the internal symmetry of the earliest divergent (conceivably the older) sister stwintrons by the accumulation of mutations (transitions, deletions, insertions, duplications) in the TIRs that do not appear to affect their capability to be excised from the primary transcript (see SRA and RT-PCR evidence in Supplementary Table S1).

Long terminal inverted repeats are typical of the most common types of DNA transposons (review of transposons in fungi [59]). The presence of blatant symmetry in most members of the family of proliferating sister stwintrons and derived canonical introns suggests that their proliferation could involve DNA transposon insertion, one of the six most commonly purported mechanisms of intron gain [19,60,61]. However, the absence of a positive bias in the exonic regions immediately neighbouring all 48 studied (stw)introns, and the seamless integration in the previously continuous exon sequences for all but one of them are both inconsistent with this model. No tandem site duplications (TSDs), which would necessarily occur during DNA transposition, are extant in any of the 48 *Hypoxylon* sp. CO27-5 paralogue intervening sequences (i.e., sister stwintrons, sheared sister stwintrons, and both types of cropped sister introns). The low-frequency intron propagation that we observe in *Hypoxylon* and *Daldinia* is therefore likely to occur by a different mechanism than the one that mediates intron proliferation described previously in two species of algae [60].

Of the seven most commonly purported mechanisms of intron gain (reviewed in [19]), both Torriani and co-workers [44] and van der Burgt and co-workers [45] argued that the mechanism of intron transposition—crucially involving reverse splicing—best fits their observations, primarily concerning the seamless integration of fungal introner-like elements in seemingly random exonic sequences. More specifically, they referred to a theoretical variant of intron transposition that involves “reverse splicing directly into the genome” [62]. This mechanism would resemble retrohoming by self-splicing group-II introns ([62] and references therein). More recently, this model of the mechanism of intron duplication was refined to involve transcription-linked R-loops as the source of the single-strand DNA (coding strand) substrate for reverse splicing catalysed by an intact spliceosome armed with an excised lariat intron RNA (from another transcript) ([41] and references therein). The model is unsatisfactory in that it does not require the involvement of the exquisite symmetrical structure of sister (stw)introns, the structural feature conserved in fungal introner-like elements [44,45,46] but lacking from the *Micromonas* introners [39,41,47].

The internal symmetry typical of the sister (stw)introns is such (Figure 6a) that it remains tempting to speculate that it is somehow involved in the molecular mechanism of intron duplication. Crucial functions of the intron TIRs could include preventing excised single-stranded RNA degradation. Here, we propose two concepts in which the internal symmetry of the proliferating (stw)intron could enhance duplication to other loci in the genome, explained with a parental type-2 cropped sister intron (Figure 6b). We presume that intron duplication can be a rare albeit fortuitous by-product of the repair of blunt-ended double-stranded DNA breaks (DSBs) by the canonical nonhomologous end-joining (cNHEJ) machinery or a competent alternative end-joining system (recent reviews on end-joining systems: [56,63]). Direct involvement of DSB repair in intron generation mechanisms at the genome level has been discussed previously [18,19,40]. The exact location of the new intron integration would be determined by the impromptu DSB, which correlates with the seamless integration of sister (stw)introns in seemingly random sequences that we observe in *Hypoxylon*. Crucial involvement of DSB repair is suggested by type-2 cropped sister intron HCOc121A where, exceptionally among the CO27-5 sister (stw)introns, intron insertion is coupled with the deletion of three exonic nucleotides immediately 5′ to the integration site (see Results Section 3.6). Thus, limited resection may have taken place before intron integration. A considerable body of evidence has emerged for the key involvement of RNA and RNA-binding proteins in DSB repair by NHEJ systems (recent reviews [64,65,66]). Excised introns can thus be actively recruited to DSBs through binding to canonical DNA repair factors or RNA-binding proteins moonlighting in DSB repair. Transient insertion of RNA stretches into DNA sequences has been reported for mitochondrial DNA and is also proposed to occur in nuclear genomes (recent review [67]).

Participating in a possible intron gain mechanism, we may postulate a crucial role of the explicit predicted hairpin structure of the single-stranded sister intron RNA (Figure 6). Self-folding would protect the intron from swift RNAse-mediated degradation, the fate of (most) regular spliceosomal intron RNAs after splicing and debranching. The folded intron RNA may then be recruited to the cNHEJ complex. The hairpin structure is such that it brings the 5′-donor G in very close proximity with the 3′-acceptor G (Figure 6a). This secondary structure could facilitate occasional self-ligation of the intron RNA into one strand of the broken DNA held together by the cNHEJ complex (Figure 6b, at the left), in a similar fashion as of the reverse splicing or retrohoming of self-splicing group-II introns ([62] and references therein). The new intron sequence would be integrated into the DNA by the same mechanisms and enzymes as proposed by Simmons and co-workers [41] for the reverse spliced introner RNA into the *Micromonas* genome.

An alternatively mechanistic model of (stw)intron propagation in *Hypoxylon* would involve an RNA-mediated version of random integration [68,69] during the repair of blunt-ended DSBs (Figure 6b, to the right of the figure). DSBs increase the frequency of random DNA integration (e.g., [70]). We propose that double-stranded (ds) RNA molecules could be formed by annealing two single-stranded molecules upon debranching of the lariats of the excised, highly symmetrical sister intron RNA. Note that the “double-stranded intron” must compete with the self-folding of the “single-stranded intron” driven by the very same TIRs. The ds-form of the sister intron, reinforced by non-Watson–Crick GU base-pairing, could act as filler RNA during the initial steps of cNHEJ-mediated DSB repair and subsequently would become permanently integrated into the DNA by the end of the repair and replication process (as proposed in the *Micromonas* model [41]). Alternatively or additionally, the recruited ds-excised intron RNA could serve as repair primers after in situ strand separation (i.e., reverting to a single-stranded form).

Remarkably, only (D1,2) sister stwintrons were encountered in the nine investigated Hypoxylaceae genome sequences, while sister stwintrons of other stwintron types and classes are absent. Xylariales species harbour a (D2,3) stwintron in the biotin-biosynthetic *bioDA* gene [9,14] as well as a (D5,6) stwintron in their reticulon-like *rtnA* gene [15] (not shown). This apparent specificity can be explained if (D1,2) sister stwintrons duplicate as one unit rather than as two entangled canonical intron units. Such an event would require the U2 spliceosome to alternatively excise the whole sister stwintron sequence (minus the G_1_ at 5′) as one longer-than-usual canonical U2 intron harbouring the TIR at each end, bypassing the canonical internal splice sites in its centre. It is possible that under certain physiological conditions, the secondary stem-loop or hairpin structure of the least divergent (presumably younger) sister stwintrons could facilitate such alternative splicing reaction [71]. Secondary structure prediction by RNAfold suggests that the imperfect symmetry in some sister stwintrons (e.g., HCOc004A, HCOc066A, HCOc070A, and HCOc178A) is considerably longer than that possible in type-2 cropped sister introns, extending beyond the 10-nt palindromes in these stwintrons (not shown). For six CO27-5 sister stwintrons, we could detect amongst extant RNA SRAs an end-product of alternative splicing that still contains the G_1_ of the external donor sequence (Supplementary Table S3). Screening the extant EC38 RNA SRAs (NCBI), we found more evidence for the existence of splicing end-products resulting from an alternative one-step splicing of the stwintron sequence, each leaving the (D1,2) stwintron’s most 5′ G_1_ exonic. Interestingly, in the gene for an arylsulfatase (606 amino acids) in *D. childiae*, the presumable generator (D1,2) stwintron has morphed into one long canonical intron (Dchc003S) from which the BP sequence of the internal intron has disappeared (Supplementary Figure S7). This illustrates the third path to create a canonical sister intron from a sister stwintron: the result being a long sister intron (195 nt: twice the size of a cropped sister intron in *Hypoxylon* sp. CO27-5) with a ~100-nt sequence between its two typical TIRs.

## 5. Concluding Remarks

There seems to be no doubt that new spliceosomal introns have appeared repeatedly throughout eukaryote evolution. Different mechanisms of intron acquisition have been proposed (review [19]). Most are supported by limited indirect evidence and, with one exception (Tandem Genomic Duplication [72]), never experimentally demonstrated in vivo. The various proposed mechanisms of intron gain are not mutually exclusive. In this article, we describe the proliferation of related complex introns and of canonical introns derived from them, in closely related filamentous fungal species from the Hypoxylaceae family, with new intervening sequences seamlessly integrated at seemingly random sequences in exonic regions in the genome. The observed proliferation correlates with elements of high symmetry in a group of sequence-related stwintrons (sister stwintrons). We observe that sister stwintrons can proliferate as such in divergent taxa but can also give origin to much shorter canonical introns with essentially the same symmetrical features, which would thus be able to proliferate as U2 introns. In parallel, new canonical introns without high symmetry can be formed by U2 intron loss from a parental sister stwintron. Thus, stwintrons may be a key intermediate in new intron appearance.

## Figures and Tables

**Table 1:** Genome sequences containing sister (stw)introns and Sequence Read Archive (SRA) databases employed in this study.

Organism	WGS Master Accession	SRA Accessions (RNA)
*Hypoxylon* sp. CO27-5	MDCL00000000 [24]	SRX875229–SRX875234 [24]
*Hypoxylon* sp. *EC38*	MDCK00000000 [24]	SRX872662–SRX872667 [24]
*Hypoxylon* sp. *E7406B*	JYCQ00000000 [25]	
*Hypoxylon pulicicidum* *ATCC 74245*	PDUJ00000000 [26] CADCWX000000000 [27]	
*Hypoxylon* sp. *CI-4A*	MDGY00000000 [24]	
*Annulohypoxylon stygium* *MG137*	PYLT00000000 QLPL00000000 [28]	
*Hypoxylon rubiginosum* *MUCL 52887*	CADCXA000000000 [27]	
*Daldinia* sp. *EC12*	MDGZ00000000 [24]	SRX872671–SRX872676 [24]
*Daldinia eschscholzii* *IFB-TL01*	AKGB00000000 [29]	
*Daldinia childiae* *JS-1345*	VYXO00000000 [30]	
*Daldinia concentrica* *CBS 113277*	CADCSW000000000 [27]	

## Data Availability

Data are contained within the article and the associated Supplementary Materials. Accession numbers for sequences determined during this study: GenBank MW477887, MW490712–MW490722, MW498245–MW498262, and MW530466–MW530509.

## Supplementary Materials

The following are available online at https://www.mdpi.com/article/10.3390/jof7090710/s1. Figure S1: Alignment type-1 cropped sister introns; Figure S2: Alignment HCOc066A–HECc034A; Figure S3: Generation type-2 cropped sister intron; Figure S4: Alignment HECc217A–HECc217B–HCOc004A; Figure S5: Phylogeny mitochondrial carrier protein; Figure S6: Base pairing TIRs double-stranded RNA molecules; Figure S7; Alignment *Daldinia childiae* Dchc003A–Dchc003S; Table S1: Contig identifiers, SRA reads, GenBank accession numbers, and more information about sister (stw)introns; Table S2: Oligonucleotide primers used; Table S3: SRA identifiers; text document S1: Fastas sister stwintrons CO-27-5; text document S2: Fastas derived canonical introns CO-27-5; text document S3: Fastas sister stwintrons E7406B; text document S4: Fastas sister stwintrons *H. rubiginosum*–*Daldinia*.
