# Supplementary material for: Internally Symmetrical Stwintrons and Related Canonical Introns in Hypoxylaceae Species
Source: J Fungi (Basel). 2021 Aug 29;7(9):710. doi: 10.3390/jof7090710 (PMC8469720; doi:10.3390/jof7090710)
Supplement: Supplementary file 1 [file jof-07-00710-s001.zip › Supplementary_material_20210825.pdf]

Supplementary Material (online)

associated with

Internally symmetrical stwintrons and related canonical introns in Hypoxylaceae species

Erzsébet Fekete[1]*, Fruzsina Pénzes[1,2], Norbert Ág[1], Claudio Scazzocchio[3,4], Michel Flipphi[1] and Levente Karaffa[1]

[1] Dept. of Biochemical Engineering, Faculty of Science, University of Debrecen, 4032, Hungary.
[2] Juhász-Nagy Pál Doctoral School of Biology and Environmental Sciences, University of Debrecen, 4032, Hungary.
[3] Section of Microbiology, Dept. of Infectious Diseases, Imperial College London, SW7 2AZ, UK
[4].Institute for Integrative Biology of the Cell (I2BC), Université Paris-Saclay, CEA & CNRS Unité Mixte de Recherche UMR 9198, Gif-sur-Yvette, 91190, France

* Author for correspondence: kicsizsoka@yahoo.com

Supplementary text document S1: Fastas sister stwintrons CO-27-5
Supplementary text document S2: Fastas derived canonical introns CO-27-5
Supplementary text document S3: Fastas sister stwintrons E7406B
Supplementary text document S4: Fastas sister stwintrons H.rubiginosum-Daldinia

Supplementary figure S1: Alignment Type-1 cropped sister introns
Supplementary figure S2: Alignment HCOc066A–HECc034A
Supplementary figure S3: Generation type-2 cropped sister intron
Supplementary figure S4: Alignment HECc217A–HECc217B–HCOc004A
Supplementary figure S5: Phylogeny mitochondrial carrier protein
Supplementary figure S6: Base pairing TIRs double-stranded RNA molecules
Supplementary figure S7: Alignment Daldinia childiae Dchc003A–Dchc003S

Supplementary table S2: Oligonucleotide primers used
Supplementary table S3: SRA identifiers

Supplementary table S1 is provided separately as an Excel file (Supplementary_Table_S1.xlsx): Identifiers, SRA reads, GenBank accession numbers and more information sister (stw)introns

Supplementary text document S1.
Fastas sister stwintrons and sheared sister stwintrons in *Hypoxylon* sp. CO27-5.

(ordered per sequence contig)

Full sister stwintrons (23)

>HCOc002A
GAATATGGACTCTATggtatgttcaggtactcagtgtctagaaagacctcaaaa**tttctaaaat**ataggaacaagctaacatcataacagtaag
taaagaattctaatatcgccgctgtccagattaaaaggcgatacttggtacatattgaaaacaaaggtcaagatgctgacacgacatgttgc
gatacactagACCACGTTTTCTGGT

>HCOc004A
ACTGGGGTGACTACAggtgagtatcaaaacacgtcgtcctggcattttagtccttaa**tttctaaaga**aaaaaaaaccctaaatttctacaacat
aggaataaactaacattatgaatagtaagttaaccgttcctatttcgttggcatcggggttaagagaaatgtctagtac**attccagaaa**ccagg
gataaaaatgctgacacgacatgtcttaatacactagTCGAGGTGAAAGTCA

>HCOc017A [mitochondrial carrier gene]
GTTTTCTGGATTACGggtgagtataaaaacatgtcgttctggcattttagatattga**tttctagaaa**aaaccccccgtttctacaatatatgaa
tcagctaacgttataacagtaagttaagcgttcttatttcgttgctatcgggattaagaaggcagtagctggtat**gttctaaaaa**cgaaggata
aaaatgctgacgcgacatgttttaatacatcagAGTTACCCAAGCGAC

>HCOc017B [other gene]
AGAGCGGGATTCTCTggtaagtgaaaaatctgccgttttggtgttttatacttcaat**ttcttgaaag**atcaccaaattcgctaacgttatgaca
gtaagtcaagtgttcttgtttcgtcgccatcggggttaagaaggcaatatctggtat**attctagaaa**cgacgtataaagtactgacacgacgtg
ttttaatagATGTTGGAGATGAAC

>HCOc021A
TTTCTAAAGATGTTCggtacctatgtattaagatatatcatgttaggatttcggcctttga**tttctagaag**aacccctatgacatagaaactag
ctaacgttatagcagtgagtaaatctttttttttttttttttcaatactatgaaacttaaaaaaggcaatttccggcgtactataagaacgaag
gtaaaaatagcactgacataatatgtttcaacacactagGGTCCTATATGAAGCT

>HCOc047A
GCCCTGGGTTGCAATggtacgtacaaaaacttcctgtggaggttttaaacccttga**cgtctgaaag**aatgtcgaataataactaatttataac
agtgagtataatgttcctctgttcctctttttaacgcctggcatagcctggaagagaaggtaaagaaaaaaaaacgaaggagagaaaaagctga
cacgatatgttttaatacctagGTTACAGCAAGTTGT

>HCOc052A
CCATCTGGCTACTCGggtgagtattaaaaacatatcgttctggcattttagacctttga**tttctagaag**actccccatatttccagtgtatatg
aattagctaacgttatgacagtaagttaagcgttcttatctcgtcgctatcggggttatgaaggtagtatctggtat**attctagaaa**tgaagga
taaaaatgctaatacgacatgttttaatacattagACTATCGCGCGCAGC

>HCOc058A
CCGTACACGAACAATggtatgtatgaagacataccgtcttggcgtattagaccttga**tttctagaaa**gaccccctgagttttcacaatatagga
ttagctaacgttaggacagtaagtaaagcgttgtcgtttcgtcatcattgaggttaaaaggaaatgtctggtat**attccggaag**caatggtcg
aaaaacgctgacacgacatatttaaatacaccagATCATTTGGGTCCAG

>HCOc061A
GCTATTGGTTCGGATggtgagcacaaaaacatgtcgttctgacattttataccttaa**tttctacaaa**cccccctatatttttacaacatataaa
ttagctaacgttatgatagtaagttatgcgttcttatttctttactatcgaggttcagaaggtagtatttagtat**attttagaca**cgaaggata
aaaatgctaacacgatatgttttaatacaccagGGCAGTACTGGTGGGC

>HCOc066A
TATGCTCTGGTGTGAggtgagtataaaaacatgtcgttctggcattttaaacctaaat**tttctagaaa**atctcccatgtttttacaatatatga
attagctaacattataacagtaagttaagcgttcttatttcgtcgctatcgggattaagaaggcagtagctggtat**attctagaaa**cgaaggat
gaaaatgctgacacgacatgttctaatacattagCCTGCGCCTGTCTAC

>HCOc070A
CTTTGTTGCTCTGGGggtaagtataaaaatatgtcgttccagcattttagaccttga**tttctagaaa**acccccctatttctacgatatatgaatt
agctaacattatgatagtaagctaagcgttcttatttcgtcgctatccggtttaaggaggcgatgtctggtat**attctacaaa**cgaaggatgaa
aatgctaacacggaatgttttaatacaccagCCGGGGTGGCTTTGA

>HCOc076A
ACGCTAGTGAAGCCCggtgagtataaaaacatgtcgttctagcattttagaccttga**tttctagaga**aacccctatatctttacaatatatgaa
ttagctaacgttataatagtaagttaagcgttcctatttcgtcgttatcgggattaggaaggcagtagctggtat**attccagaaa**cgaaggata
aaaatgctaacacgacatgttttaatacaccagTGCTGCAGAAGGTGG

>HCOc102A
CATTTAATAGTCAGTggtgagtataaaaacatgtcattctggcattttaggccttg**tttctagaaa**acccctcatatttctacaatatatgaat
tagctaacgttatgatagtaagctaagcgctcttatgtcgttgctatcagggttagggaggaaggataaaaatgctgacacgacatgttttaat
acaccagTTCAAGAAGCTACAA

>HCOc164A

TAAGGCAACTACAACggtatgtattaggtagatatatggtgtggtgggattccggcccttg**cttcctagaaa**agccttcatttctctatgatgc
aggaaccagctaatttttatgacagtgagttaaaacactttattttgtcgccgtcgaacttgtgaagagaatatcaagta**tatcctagaaa**tg
aagactatagatattgacactacatgttttaacactttagGGAAAGGGAGCGATGT

>HCOc178A
CCTAGACCACCCTTCggtgagtataaaaacatgtcattctggcattttaaacattga**tttctagaaa**accccccatatttctaccgtatatgaa
ttagctgacgttgtgatagtaagttaagcgttcttatttcatcgctaccgggttaagaaggcggtatctggtat**attctagaac**gaaggataaa
aatgctgacacgacatttttaatacaccagGGATATCAGCGAGCAT

>HCOc236A
CGCGCTTAGCATCTTggtgagtatacgaatatgtcgttttgtcactttagaccttga**tttctagaaa**gatcctcaaatgtcttcaatgtagga
attagctaacgtcgtgacagtaagtgaagcgttcctatttcgtcgccatcggtgttaagaaaacaatgctaggta**tatcttagaaa**cgaagtgt
taaaatgctaacaagttatgtttaattacaccagCGCGCTTAGCATCTT

>HCOc224-179 [split over contigs 224 and 179]
[GenBank Accession number MW477887]

ACGGACAGCGCCTCTggtgagtataaaaacatgtcgttctggcattttagaccttga**tttctagaag**accccgacccccccccccaacatattt
ctacaagatatgaattagctaacgttatggtagtaagttaaacgttcttatctcgtcgctatcggggttaagaaggcagtatctcttat**attct**
agaaacgaaggataaaaatgctaacacgacatgttttaatacatcagTCTGTCCAACATGCG

>HCOc252A
AGGGTTGGCGTCCCAggtacgtataaactatgccatttcggcattttagatcctggtccc**tacctagagg**tttagctaacgttaagacagtaag
ttgatcgttttttatttcgttattgtcgaggttaacaaggaaatgtttggttt**attctagaat**gaagtattgaaaaggctaacacgatatgttgt
aatatcctagGGATGGAGGGATGCAA

>HCOc271A
ATTGGATACGTAATTggtgagtataaaactatgtcgttttggcgttttagacatttgg**tttctggaaa**gaccatcaaatctcaatataaaaatc
agctaacgttatgacagtgagtcaaatgttcttatttcgtcgcgatcggggttaagaaggcaatgcctggtata**attctagaaa**cgacaggtaa
aaatgctgacacgacgcgttttaacacgttagTTATATGCCGCATTG

>HCOc332A
GGTGGATGCTACAGTggtatgtataaaaatatgtcgtttggcatcttggagactgg**ttttcataaa**atatatatgaattagctaacactgggac
agtaagtaaaccgttctcatttcgttgctatcgagtccaagaaggcaatgtctagtat**attctagaag**caaaggccaagaatgctgacacgaca
tgttctaatacaccagAATCGAACTGAGAAT

>HCOc378A
GAAAGGGGTCATATTggtgagtacaaaagcatgtcattctggcattttagaccttga**tctctaagaa**aaccactatatttctataatatattaa
ttggctaacgttatgacagtaagttaagcgttcttatttcgtcgctatcgcggttaagaaagtagtgtttggta**tattctagaaa**cgaaggata
aaatgctgacacgacatgttttaatacaccagTCTTTACCGAACTGA

>HCOc406A
TGGCGCAAGACTGTTggtgagtataaaaagacgtcattttggcatttcatacccaga**ttcccacaat**ataggaattcgctaacactatagaagt
aagttgagcgttttatctcgtcgtcatcggggttaagaaggcaatgcctacctagtat**attctagaaa**tgatgggtaacaatgctgacacgac
atgctttaacacactagTTACATGCCATCGAC

>HCOc522A
CGACGCCCCCGACATggtatgtataaaacatgttgccttggatttccaagcattga**tctctggaaa**gactcgtaaatttccatagtttaggaat
tagctaacgttgggatagtgagtaaagaattatcattttatcgccatcgagtttaagagcgcgacgacctggtaa**agtctagaaa**cagaagtca
aaattgctgacaggatacattttaatacactagAAAAGGTGGCATGAC

sheared sister stwintrons (12)
patches of homology with full sister stwintron HCOc017A (yellow and underlined)

>HCOc002B
AGTGGCAGATTAATCggtacgtataaaaacggcaagagatattcatatttcagtccccagattttcgaacatccttcaaattttcacaacgcaa
tagctaacattgtgaaagtgagtagaaatatcgttgccttactatcaaatttgcgagggcaacgatctgttccctccatatgagaattttacaa
tgctaacatgacatattttgatacatcagCTTCTCCTGGAAGTA

>HCOc004B
TTTGAGATGCCGATTggtatgtaggaagacgcgtctcgtcgttattctaactccgactgtcaaatgacttctaaatttctgtatagtagtaact
aaccttctagtagtgagtcgaatatcctcgttttatcctccccaaacttaagatggaaattcccagtat**attctagaaa**tagatgttataatac
tgatatgacgcgtttcatacaccagCTTCTAGGGAAAGTG

>HCOc016A
TCGGAAGCTTAATATggtaagtatccctacataacctgtcctgttctcagcttatagttcttgatccccttctgttcagtagtcaatcaaacgt
gtagctaacattcacttagtacgtacaggatcttcttcctcttcaatcattttctataacaattaaatgcctaggagacacctcaaacggtcca
aggtaaacgttgttagattactaacgcgacatgatgtaatatgacagATCGATACACTGGCG

>HCOc016B [other gene]
AGAGTATATCCGTGAggtgcgttcatttctttatctccattcatccttgctcatacttcatttatatgcatgcatacgctaacgataggacagt
aagtaaccaagatttcctaacctcatcttcaggcttagatctccttcctggagaaacctacgagaggctcctttttctaactctataaagATAAT
TGTACAGTCG

>HCOc020A
TCCTGCAAGTCATCCggtgcgtgtaagaccatctcgtatcttaattcttgtccttgactatttaataattttaagattcgtataggatagtaac
taactatacggtagtgagtgaaacatttgtttattaacactgaatctagaaaagaaaagcaacttctagtgataggaaaggagttagaagtact
gacacggtatgtttcaataaaacagGGGTGAAGATTCCACA

>HCOc024B
ATCTCTTTTCAGCTCggtatgtagtagatggcctcaatttcggattccataccccttttatctcttcgttcactcttcgtactaacttgtgg
atagtgagtgactaattcaccttctacctattctacggattaagggctcaatgaacgatttgagatactaacgtattgcagAAATCTTCAAATG
GG

>HCOc046A
GGAGTGACCGATTGTggtatgtacgaaaacatctcgtttgagcatttcaaagcccct**tttctagaat**aatcttcgaacttctcgtacatccatt
gctaactccccatagtgagttataacactctaagacgttcgagatgatggtgatataatgctgacacaagatgttttcctccaccagACGACAA
CGTACTAA

>HCOc091A
ATGGATTCAGTCATCggtaagtattagagcaagggatcatatttctgaacccttcagccccatttcatatttgcctaccccgattgactgtcgg
taggtaattagctaacctcctcatagtatgttgaactatttcttccgttcaccgttctattactatagtgagctatgaaataagttataagatt
actgacgaacaattttatccatgttagAATATTCGACGCCAG

>HCOc091B [other gene]
GCTGGGCGAACACTTggtacgtagtgtcgtgtcgagatttcaagagctatctttcgaacggtctccaattctctttggtatagataaaagctaa
cttcctggcagtgagtaaaatgatcctattccatttgcacgatatttattcggtatgtactaaaagtaaatgacgagatactgacgtgccataa
tatactagATGATTCGCTTGAAT

>HCOc159A
TCTGGTTTATGACTTggtacgtatgaagatatgccatgttacagtttgaacctctagccccttgaaagatctcctagtttattcaatacttcag
ctaacgttacggcagtaagtgaaacacattcatttctccgctgctcaatttagaacagcaacgtctagcgttttagagaaaagagatatggcat
actgacatgatatactctggtattaagGGTTAACCCGGACGG

>HCOc263A
TCCTGCCACCCACCCggtacgtctaatcccatatcgtgtgatatcgaatgatctccgtgttcctataatacagtactaacgctcagacagtgag
taacatcgagttctttatttcccctgtcgacctgtcgcgtgggccagcgtttccaagttaggaggaatgtggaatggtcacccctaaaacatgc
ctaaaactcccgaacaccttggaaaaaatcatcactaactcttcctacagCGTCCCAGTCCCTCA

>HCOc304A
AACCTTTGGCCAATCggtacgtatagatgcatttctagagtatctttcaattcttctcacacgcaaacgctaacatttcacagtaagttgaata
attctgctatgccccaagcttgctacggatcccctagatgcactcgagctaggggaatatgatggtagcccttcatggctaggctgattgctga
cacaacacgctttaatacaatagTTTTACTCTGCCAAT

half-sister stwintron (1)

>HCOc103A
TGTGAGGAGATGCTTggtatgtatcaaaatatgtggattcaggattttgaaatttggtttttacaagaatgttttgaaatttggtttttacaa
gaatgttttgaaatttggtttttacaagaattttcgaatagctataatatagtggaaagctaacgttagaatagtatgtcaaacacccttatt
tcgttgctaccaagcctagaaagcaccgcctggtat**attctagaac**acgaatcttaaaattaggtactaatactatagttgtgtctaaaaaaaa
acagACAGACCAGAGCGGG

Supplementary text document S2. Fastas cropped sister introns in *Hypoxylon* sp. CO27-5.

HCOc047B (1) and HCOc229A (1) are the two type-1 cropped sister introns.

Clade of related type-2 cropped sister stwintron with the extra G near 3’ marked magenta: The marked T is A in all other sister (stw)introns.
(ordered per sequence contig)

>HCOc024A (2)
TGCGGACCATGCTCTgtcagtataaaaacatgtcgttctggcattttagacctcaa**tttctagaaa**cgaaaaataaaaatgctaacacgacatg
ttttaatagGGGCCGCAATGCAGG

>HCOc047B (1)
AGTATTCGAGGTTTGgtgagtataaaaacatgtcgttctggcattttcgacctcaa**tttctagaaa**aatcggccgtatttctattaaaaaatat
gaattaactaacgttataacagGTTGCATAGGGGCAT

>HCOc096A (2)
CGATGATGTCCTAATgtgagtataaaaacatgtcgttctagcattttagaccttaa**tttctagaaa**cgaaagataaaaatgctgacatgatatg
ttttaatacactagCGCGGCTATCAGACA

>HCOc100A (2)
CAGGAGAGTTTTAACgtgagtataaaaacatgtcgttctggcattttagaccttaa**tttctagaaa**cgaagtataaaaatgctgacacgatatg
ttttaatatactagTCGAACGGAAACCAG

>HCOc105A (2)
CGAGACGATGCAGTGgtgagtataaaaacatgtcgttctggcattttagaccttgt**tttctagaaa**cgaaagataaaaatgctgacgcgacacg
ttttatacacgcagAAACTAGACCGCGAG

>HCOc121A (2)
TCGCATCAGCTACTGgtgagaataaaaacatgtcattctggcatattagaccttag**tttctagaaa**cgaaagataaaaacgctgacacgacatg
ttttataccgcagACCAAGCCTACGCAC

>HCOc147A (2)
CACCTCGCCTTCCGTgtgagtataaaaacatgtcattaaggaattttagaccttaa**tttctagaaa**cgaaagataaaaatgctgacacgacata
ttttatacacgcagACCACGGACGCGTAC

>HCOc153A (2)
ACATGCCAATTGAAGgtgagtataaaaacatgtcgttctggcattttagaccttaa**tttctagaaa**cgaaagataaaaatgctgacacgacatg
ttttatacacgcacagCTCTACGACGAAGCC

>HCOc164B (2)
GAGACACGTCCTATCgtgagtataaaaacatgtcgttctggcattttagaccttaa**tctctagaaa**cgaaagataaacaggctgacacgacatg
ttttaatatactagCACTAGGACCGTGTT

>HCOc171A (2)
CTCGGCTATATATTGgtgagtataaaaacatgtcgttccggcatcttagaccccaa**ttcctagaaa**cgaaagataaaaatgctgacacgacatg
ttttatacacgcagGGACACTCCAATCGC

>HCOc229A (1)
TGGATATTGCCTTTGgtatgtatgaaaacgtgacatttcgacatttagatttgaatctgaa**aaagatcctt**aaagttctattataccagacctag
ctaacgttatgacagACGTCCTTTCGCTAG

>HCOc343A (2)
TGTTTTCGGCCGGTTgtgagtataaaacatgtcgttctggcattttagactttaa**tttctagaaa**cgaaagattaaaatgctgacacgacatgt
tttatatacggagATGGATCGTGGTACG

Supplementary text document S3.
Fastas sister introns and the type-2 cropped sister intron in *Hypoxylon* sp. E7406B.

includes one sister stwintron unique to *H. pulicicidum* (i.e., absent from E7406B)
(ordered per sequence contig)

>HE7c016A
ATCTGTGTGATGACCggtacgtatgaaaatattcccagatttagataatgtagacgtcagctaacatttaagcagtaagtaaagcattttcatt
tcgtctccatcgagcttaaagaagcaacgtccggtat**gttctagaaa**cgaaggcctaaaatgctgacacgaaacgttttaatgcattagATAAC
CCACAAGACG

>HE7c026A
GCCAAGTCTCGATTTggtacgtataaaaacgtgtcgtcccggcattttagcccctga**tttctagaga**taccccctatacatacctacctcccatg
tattggctaacattaaacagtaagtagagcattctcattccgtcgccaccgagcctaaagaagcaacgtccagtac**gttctcgaaa**cgaaaccc
ccaaaatgctgacatggcatttttcaatacattagGCCGGTACTCCTTGG

>HE7c026B
GAGATGGCATCAGGAggtacgtgtgaaacatgccgtgccggtattttagcccttga**tttctagaaa**tatacccggatttgtataacgtaggcat
tatctaacatcaaaatagtaagtgaagcattttcattcgatcgctatcgagcttagagaagaaatgttcgatat**attctagaaa**cgaaggccga
aaatgctaacatgacacgttttaatacaccagCCCAGGGTGGTTTGAA

>HE7c035A
CCCCAAAATACCTATggtatgtacaacattatgccgtgttggcactttggtccttgtgttgtagttaagtccctaaa**tttctataat**atgagaa
ttagctaacgttatgacagtaagcagagcaaccttattttcattatcgagcttaagaagaccataaccgatat**attatggaag**cgtaggtac**c**
tagagagttaaaagcgctgacacgatatcttttaatatgctagAAACACTCCCGCAAA

>HE7c050A
TCGGCATCATCACTTggtatgtataagatcaagtcgttccggcatttagcccctgatttccatggtatagacattagctaacattgtggcagta
agcaaaacacttttattccgttgccatcgagcttaagaaggcat**atcccagaaa**ctgaggtaaaaatgctaacccgacgtgcttttagATAATT
GGGTGAAGC

>HE7c129A
AGGTGGCACGATTATggtacgtataaagatatgctgtcctggcattttaacccctg**attttagaag**tatccctacatttagatattatagggat
tagctaacattaaacagtaagtagagcattctcattccgccgccatcggacttaaagggacaacgtccggtat**gctcttgaaa**cgaaggttaaa
aatgttgacgcgacgtgttttaatacattagTTCCGGCTACATTAT

>HE7c137A **[unique to E7406B]**
CCGTAACTGGAGATCggtacgtataaaaatgtgttgccccggcattttaacccctga**tttctggaaa**tatcattatgtttacgtagtgtaagca
tcggctaacgttaaacagtaagtagagcattcttgttccgtcaccgccgatcttaaagaaacaacgtccggtac**gttctagaaa**taaaggttaa
aaatgctgacacgacgtgttttaatacactagTTTGGGTAGTAAGGT

>HE7c276A
CTACCCCGATTATGCggtatgtatggaaaatatgtcgtcccggcatttcagccctttatttttttatttttat**tttttagaaa**tacccccaggt
ttattatataaacgttaactgacatttaaacagtaagtaaaccatttccgttcggtcgccatcgagcttaaacaagtaacgtttggtat**attct**
aaaaacgaaggctgaagatgctgacacgacatgttctaatccatcagCTATGACTTTTCCGG

>HE7c301A
CAGTCAGGGTCGCGTggtacgtatggaaaagtgccttcccggcattttaacctctga**tatccagaag**taaccctagatttatatagtacgggca
ttagctaacattaaacagtaagtagagcattctcattttgtcgccgtcaagcttaaaggagcaaggtccagagt**gttttgaaaa**cgaaagctag
agattctgacacgatatgtcttaatacactagCTACATTACAGCAGA

Type-2 cropped sister intron *Hypoxylon* sp. E7406B & *H. pulicicidum* MF5954

>HE7c057A
CTAGTTGGTTCAATGgtacgtataagaatgtgtcatccaggcattttagcccctaa**tctctagaaa**tatccctaaaattatataatgtaggcat
tagctaacattaaacagATGATGTCGGCTGTG

***Hypoxylon pulicicidum* MF5954 [unique]**

>Hpuc023A
CAGCGTGATCATTACggtacgtataaaaatgtgtcgtcccggcatttcaaccaccga**tttctagaaa**tatacctatgtttacgtagtacaggca
ttagctaacattaaatagtaagtagagcattctcgttccgttaccaccgagcttaaagaagcaatgtccggtat**gttctagaat**caaaggttaa
aaatgctgacacggcatgttttaatacactagGATATTCTAGCTAAG

Supplementary text document S4. Fastas sister stwintrons in *Hypoxylon rubiginosum* and the *Daldinia* genus.

[*Daldinia childiae*, *D. concentrica*, *Daldinia* sp. EC12 and *Daldinia eschscholzii*]
(ordered per sequence contig)

***Hypoxylon rubiginosum* MUCL52887** (5)

>Hruc29A
TAACTTCATGTATTTggtacgtaataccttgtggtaacttaaagattatcacatctccccgaccctccaagaatggtcagggtaaagctatctc
gaagattctagataacctgagaagaattaaaggggtaaaagaagaagttttctgcctagaagctacttgcactttctcgaatactaaaaaaaa
tgtcgacgtgggatgtttttatatacgtagtacgtatgaaaacatgttgtgtcagcatttttattgcccc**tttctagaat**aatctcgagtttat
atgatatatagtaatttcgctaacattctaacagtaagtattctagtttcttcgctgtcgagttgataagagcagcgtctggtatgttcgagaa
acctgcgttaaaaaatgctaacacgacatgttttcgtacactagACACCGGCCAAGGGT

>Hruc31A
GTGCGCTGGGGCCATggtatgtatgaaaacatgtcgtgtcggcatttttatcgtccctttctagtataatctcgagcatatataatatggtaat
ttcgctaacattctaacagtaagtattctagtttctccgctgttgagttgaagagagcaacgtctggtatgttcgaaaacctgcgttaaaaatg
ctaacatgacatgttttcgtacaccagATACAACCTCGAATG

>Hruc55A
TGTTTTTTTATTTCGggtatgtatgaaaacatgtcgtgtcggcatttttatcgtccctttctagtataatatagtaatttcgctaacattctaa
cagtaagtattctagtttcttcgctgttgagttgaagagagcaacgtctggtatgttcgagaaacctgcgttaaaaatgctaacacgacatgtt
ttcgtacactagGAACGACCATCTTCC

>Hruc56A
TGGAACCAGGTCGTCggtatgtatgaaaacatgtcgtgccggcatttttatagcccctttctagaataatctcgagcttatataatatagtaat
ttcgctaacattctaacagtaagtattctagtttctttcttcgccgtcgagttggagagagcagcgtctggtatactcgagaaacctgcgttaa
aaattgctaacacgacatgttttcgtaacaccagGAGCAGTACTCGCAG

>Hruc59A
AGACGACGCGGTACTggtatgtatgaaaacatgtcgttgtcggcatttttttatcacccctttctagaataacctcgagcttatataatattttt
gctaacattctaacagtaagtaattctagtttccttggctgctgcggtctggtatatttcgagaaacccaacgttgaaaatgtgctaacacgac
atcttttcgaaataaaaagGGGATTGCTGTAAGC

***Daldinia childiae* JS-1345** (18)

>Dchc001A
TTGAAGGAGACGCGTggtatgtatgatatacgtttcgtagtattatttccaaccatccc**tttctaaaat**agtctcaaatctgcctttaggaggt
aacagtagctaaccctcggtagtaagtgtgaaagcctatcatttccactcattcgaaaaaggacgatggag**attctaaga**ttctaagattgcaa
agctgacttggaatattttcatacataagATGGATATGTGGTCC

>Dchc001B **[same gene]**
CTGGACCCTCAATAAggtatgtacctaatgaaaacattccgttttgctgttttcgaaccttctttttagaattgtcccgagttatagttgctaa
cgatcaatagtaagtgtgacgaccctataatttcgactcgcccaaggttatg**attctataat**cgtatgactacaaaactaatacaaaatgttcc
caaatagTGGAGGGGTAGGTTC

>Dchc001C **[same gene]**
GGAGCTAGCCTTCGTggtaagtacaatgaactcc**tttctaaaat**tgtttcgaaatatagttgctaacagtcagtagtaagtgtgacgatcctat
aattctgacctactagaatacgatgaag**attctaggat**cattggttatagagctgacatgtaatattttcacatattagTCTGATGGCGCGCCT

>Dchc001D
AACCATCTTCAATATggtatgtatgaaaacatccctttttatagagtggtttcgaaattatcttgataagatacctatacagtggctgacgctca
gcagtaagtgcgatcgccctatcatttcgacctaatcgaacccctttctctctctcccccccccccccccccccccccccccggtgaag**attc**
tggaagtgtagtatcgcaaagctgacgtaaaatgttttcatacatcagAGCTCAGTTCCAGGA

>Dchc002A **[half sister stwintron – external intron]**
CCAAGGTGGATATGTggtatgtataaagacgagacatgttacccctggcccttcttcaacctacctctctgttctttcatgtatagttgctaat
aattagtagtaagtagaagatccctgttctcccatcatctggcaaagaacattcaaactcctgtaaaggtaggatcgcgaagctaacactagat
tttctcatgcattagCAACACGCCGGGAGG

>Dchc003A **[see Fig.4]**
ATCATGCCTAGCAATggtatgtatgaaaacatcccgtgttattcttttgaaaccccc**tttctagaac**ggtttcggaaccacctaggtatacagt
agctaacgctcagtagtaagtgtgatggccctatcatttcgactaaataaaaaaagaacagtgaag**attctagaat**tgtagtaatgcaaagctg
atacgagatgttttcatacattagGTCGATCCAGCGACC

>Dchc003B
TAGAGAAACCACTCCggtatgtatgaaaacatcccgtgttgttattttcaagcccccc**tttctagaaa**ggtctcgaaactatcctcgtaaggta
tacagtggctaacgctcagtagtaagtatgatagccctgccatttcgacccaatcgaaaaataacgatgaag**attttagaat**tgtagtattgca
aagctaacgcaaaatgttttcatacattagCAATCGGCTTCAGAG

>Dchc003S [paralog 1: lacks internal PB-A]
CGACTCATTCATCTGgtatgtatgaaaggatcctgtgttgttattttcgagccccttttccatggcagtctcgaaactaccttcgtaaggtata
tagtggctatcagtagtgtgatggccctatcatttcgacgaaatcgaaaaattatggtggaa**attctaaaat**tgtaatattgcaaagctgacac
taaatgttttcgtacatgcagGTATGGTCCACGATG

>Dchc003T [paralog 2: lacks internal PB-A]
ACTTCCAAGAGACTGgtatgttcgattcgtacactatgtatcaggaggcgttgaagcagaggaaagcggagaaagtgagagtgaaagtggacac
cgatcagtagtatgtatacatatccagagcattggagctcaagtcaactaacctattcatcaagcagTCCGCCATATGTTTC

>Dchc004A
GGTGCGGTCTCCGTCggtatgtatcaaaaatactctatgcttgttattttgaaacccccc**tttctataat**agtctcgaaaccgccttcgtaaggt
atacagtggctaacgtttagtagtaagtgtgatagccctatcatttcaacccaatcaaaaaaaaaataacgccaaag**attctagaat**tgtagta
tcgtaaagctgatacgaaatgtttgcatacattagATTGATACTTCGGAT

>Dchc004B
AACACGCATGAATCCggtacgtgtaaaaatactctgtcgttgctttcaacctccct**attatagaat**gacacctacactacctcctttgggtaca
cagtgactgacgtttaatagtaagtgataaccttatcattttgacttaaaaggaacaggaaag**atcctataac**tataggattgtaaagctgata
cagaatgatttatatatcagAAATATCTGGTGCGA

>Dchc004C
AACCTGGCTACGCTTggtatgtattaaaatgtcccatatccttattttgttagccct**attccaaaat**attcccaaactgcattcattaggcata
caatgactaacgctcagtagtaagttcgacaatccctattatttgactcgtttgtaaaggagtcgtgggt**atcctagaac**tagaggattgaga
atctaatgtggaatgttttcatatattagATACCATTGACGTTG

>Dchc005A
AGACACAAATCCATTggtatgtatgaaaacatcccctgttgttattttggactctc**tctctataa**tggtcttgaaatatagtagctaactctc
agtagtaagtgtgatgacccccctcacttagagtcactcgaacatggcgaag**actctagaat**ttgcagggttgaaaagttgacacgaaatgttt
tcatacattagCAATGGATGGGCGAA

>Dchc005B
GTAGCGGTAAACCCGggtatgtattgacaattatatattgtaatatccaaaatctcctttcctatatcgtttcaaacatttatttcatctacat
cggctaacgaatagtagtgagtaggattgtcctattatttcgacttactcaagaaataagtttgaat**cttctagaat**tataagacctcaaagct
aacacaagatctttcggtttgctagAAAGACGTGAAGGAA

>Dchc007A
CTGGAGCTCCTCTTTggtacgtacgaaaccatcccgtgtttattttgaaacttcc**tttctagagt**agtctcaaaattgccttcgtagagtatac
agtggctaacgtttagtagtaagtgtaacgaccatatcattcgactcaatcgaaagataatggtgagg**attctagagt**tgtagggttaaaaagc
tgacacgaaatgttttcatacctagATGCGAAGCATGGGT

>Dchc007B
ACATGGGCTTGGTACggtacgtattaaagcatctcatgttgttattttcgactctcc**tttttagaat**gatctcgaatacaatggctaatgttta
gtagtaagtgcgatggtcctatcacctggattctctcgaacgcgatg**atcctagaat**tgtagggttacgaggctaacacgatttcataaattag
GCGTACCGTGGGTAT

>Dchc011A
CGGGATTATTTTTATggtatgtatgaaaacgccccacgttgttactttcgagccattc**tttatagaat**ggcctcgaaactacttccctaaggta
tacagtggctaacgctcagtagtaagtgtgattgacgaccctatcatttcgacccaatcgaaaaaaaaaaccaacggtgaag**attctggaat**c
atagtattgcaaagctaacatgaaatgcttccacgcattagACGGTGCTGCAGCCA

>Dchc013A
GGTGCACAGTTTTACggtataaaattaatttgaacccc**cctttagaat**gtatgcaatcgctaacgttcagtagtaagtcatgatgatcttatca
tttcgactccctcgaaaagaaaacggtaaag**attctggaat**tacaagaatgtaaagctgacaaaagatatattcgtatatatagATGTCGTGCT
ACCAA

***Daldinia concentrica* CBS 113277** (9)

>Dcoc02A
CTGGAGCTCCCCTTTggtacgtatgaaaacatcccgtgttgttattttcgaatcccc**tttctagaat**ggtctcgaaaccaccttcttaaggtat
acagtggctaacgctcactagtaagtgtgatggtcctatcatttcgactcaatcgaaaaataacggtcaag**attctagaat**tgtagtattgcaa
agctaacacgaaatgtttcatacattagATGCGAAGCATGGGT

>Dcoc02B
TATTAATTTAATCACggtatgtataacccccctacctcctagaatggtttccaagtatacagtaactaacactcagcagtaagttgcgacgacc
ctatcatttcgactcactcgaaaaagaacagtaaaa**attc**cg**gaat**tgtaaggttataaagctaacacaggatgttttcatcagTTATTATGCA
CCAGT

>Dcoc03A
GACACTAGAAATTATggtatgtatgaaaacatcccgtgttatt**attctagaaa**tatctttcgagaatagtctcggaatcatcctcgtagggtat
acagtagctaacgctcagtagtaagtttgattatcatttcgactcaatcgaaaaatattggcgaag**attctacaat**tatagcattgcaaaactg
acacaactgacacaatgttttcataacttagGTATGGTCCCCGTAC

>Dcoc05A
GTAGCAGTAAACCCGggtatgtataaataattatgtattgtgacctccaaaactccctttatcggatagtttcctagatttcttttatgtacaa
tggctaatgcatagcagtgagtgagatgatcctattatttcgacttaagaaataattttgaaa**cttctagaat**ggtaggatttctaagctaaca
caagatttctcgggttggtagAAAGACGTGAAGGAA

>Dcoc06A
TTTGATGGGACTATTggtatgtataaaaaagcatttcgtatgatt**gtt**t**t**c**gaaa**tgtttgacttgcagtagctaatagttaatagtaagtggg
ataaactctgtcatttcgactctctcaagagaatgtcgaagtatatatacgtacaacggtagggttgcaaagctaatacaagatgcttcgatat
actagATGGCATCCTCACAC

>Dcoc11A
CCGGAATTAACTTCAggtatgtataaaaatgacccgtttattacttcccaacccctattccttcgtaaggtatacagttgctaacatttagta
gtaagtgccatcacttaataaaaaaaagaacgatgaag**attttagaat**tataaagctgacacagaatgttttcacgcattagTTTTCTACTTCG
GAA

>Dcoc20A
GGCGCACAATTCTACggtatacaattaacccattctacttttaacccccctttaggaggtttacaaccgctaacgcccaatagtaagtggtgat
gatcctatcatttcgactcgctcgaaaaagaaaagaacattaag**gttctagaat**tacaggattataaggctgacgcaagatgtattcgtataat
tagATGTCGTGCTACCAG

>Dcoc38A
TCAAAGGAGACCCATggtacgtctgatatgcgtcccatgttgttattttcaatcatccc**tttctaaagt**gatctcgaatccgcctttataaggt
atatagtggctaacgctcagtagtaagtgtgaaagtcctatcatttcggctcattcgaaaaaggattgtgaatattttaagacgctaggattgt
gaagctgacacagaatgttttcatacataagATGGCTATGTGGTCC

>Dcoc41A
AGCCAGGTTACGCTTggtacgtatgaaaacgtcccatgctgttattttcaaatcca**attctagaac**agtctcgaactgcattcattaggtttac
agtagctaacgctcagtagtaagtgcgatgaccctatgatttcgactcgctggaaaagggaccttgggt**attctacaac**tagagggttttaatg
ctaatacggaacgctttcatacatcagATACCATTGATATTG

***Daldinia* sp. EC12** (7: two are identical)

>DECc253A
TTCCATCATGCTGGGggtacgtattaaaacatccttctttgttatttccatatcgccattatatactgctttagaaactgcctttataagatgt
gcaatagctaactttcagcagtgagtcacgatgaagtcgtatattgacactcgataaggaaccgtgaaggtttcagagtagtaaaattgcaaag
ctgacacgagatgtttttcttatattagATGGAAAGAACCTCG

>DECc262A
GGACACAAACCCCTTggtacgtatggaaacgtctcatattgttatatttaaatcatcattcctaaataatcaagcataaatagctaactttcag
tagtaagtgcggtggtatctattaaaccactttaaaaaggatggataaggttctagcgatgtaggattgcaaagctaatacgagatgtttacat
atactagCAACGGCTGGGCAAA

>DECc304A **[identical gene: twice in the contigs]**
CTGGAGCTCCTCTTTggtacgtacaaaattgtcctgtattgttattttcagaattgt**attctagaat**ggtattaaatagctaacgtttagtagt
aagtttactagcccctatcaactgatccactcgacaaggaacggtaaagattttggagttataggattgtaagactaacaatagattcatatat
tagACGCGAAGTATGGGT

>DECc498A **[identical gene: twice in the contigs]**
CTGGAGCTCCTCTTTggtacgtacaaaattgtcctgtattgttattttcagaattgt**attctagaat**ggtattaaatagctaacgtttagtagt
aagtttactagcccctatcaactgatccactcgacaaggaacggtaaagattttggagttataggattgtaagactaacaatagattcatatat
tagACGCGAAGTATGGGT

>DECc320A
GCGGCAAATTCACCTggtatgtatacaaatgtcattctagaatactctttaaaactgtccttgcaagatatacgatagctaacatttagtagta
agtttgataaccttatatatcgactgccgattaagaacgatgaaaatttcagaatagtatgattgtaaagctgacacgatatgttttaacacat
tagACTGGGAAAAAGCTG

>DECc413A
GTTAGCACACGATATggtacgtaagaagaaaaaaaaaccatccagcgtgctatttctatgccccttttccataatcatctcgaaattgtctttg
gtatacagtggctaacgttcagtagtaagtttcgacttaatcgacaaagaaccgtggag**attctagatt**tgtagcaacacgaagctgacgatga
aatttttacttatcagAACCAACCAAGTATT

>DECc501A
ATTGAGGAGGCTTTTggtatgtataaacacgtcccgcattgttatcttcgtaaccta**atttcagaat**ggttctaaagctgcttttatataacta
agctttacgattgctgaccttcagtagtaagtgtcatcgtcctgtctattgacggactcaaaaagggagaatatgatagagctacaagagtgca
cagctgacttgggatgttttcatatactagAGCGAGAAGGTTATT

***Daldinia eschscholzii* IFB-TL01** (6)

>Desc187A[= same location as DECc501A]
ATCGAGGAGGCTTTTggtacgtataaacacgtcccgcattgttgtttttcgtaacctaattctagaatggttctaaagctgcttttatataact
aagctttacgattgctgaccttcagtagtaagtgtcatcgtcctgcctattgacggactcaaaaagggagaatacgatagagctacaagagtgc
acagctgacttggagtgttttcatataccagAGCGAGAAGGTTATT

>Desc198A [= same location as DECc253A]
TTCCATCATGCTGGGggtacgtatgaaaacatcctgctttgttattttcttatcgccattctatactgctttacaaactgcctttataagatgt
gcaatagctaacgtttaatagtaagttacgatgacgtcgtatattgactctcgataaggaacggtgaaggtattagagtagtaagattgcaaag
ctgacacgagatgtttctcttatactagATGGAAAGAACCTCG

>Desc274 [= same location as DECc304A & DECc498A]
CTGGAGCTCCTCTTTggtacgtacaaaattgtcctgtgttgttattttcagaatttcattctagaatggtctcaaatagctaacgtttagtagt
aagtttaccaacccctattcactaatttattcgagaacggtaaagactctattgtaagactaacaatagattcgtatattagACGCGAAGTATG
GGT

>Desc420A [= same location as DECc262A]
GGACACAAACCCCTTggtacgtatggaaacgtctcgtattattatatttaaatcatcattcctaaataatcaagcattaatagctaactttcag
tagtaagtacagtgatatctattaaaccacttaaaaagggatgggtaatgttctagcgatgtaggattgcaaagctaatagggggtgtctcata
tactagCAACGGCTGGGCGAA

>Desc618A [= same location as DECc320A]
GCGGCGAATTCACCTggtatgtataaaaatgtcattctagaatacgctttgaaaatgcccttacaagatatacgatagctaacgtttagtagta
agtgcgatgatcttgtatatcgactgccgataaggaacggtaaagattttggaatagcatgattgcaaagctgacacgatatgttttaacacct
tagACCGGGAAAAAGCTG

>Desc640A [= same location as DECc413A]
GTTAGCACACGATATggtatgtataaaaatgtcttggattgttatttccatgccccttttcataatcatctcgaaaattgtctttatgatgta
tacagtggctaatgttcagtagtaagttttgacttagtcgacaaagaaccgtggagattctagatttgtaggaacacgaagctgacggtgaaat
gtttacttatcagAACCAACCAAGTATT

```
HCOc004A [Int]      GTGAGTATCAAAACACGTCGTCCTGGCATTTTAGTCCTTAA-TTTCTAGAAAAAACCCTAAATTTCTACAACATAGGAATAAACTAACATTATGATAG
HCOc017A [Int]      GTGAGTATAAAAACATGTCGTTCTGGCATTTTAGATATTGA-TTTCTAGAAAAAACCCCCCGTTTCTACAATATATGAATCAGCTAACGTTATAACAG
HCOc076A [Int]      GTGAGTATAAAAACATGTCGTTCTAGCATTTTAGACCTTGA-TTTCTAGAGAAACCCCTATATCTTTACAATATATGAATTAGCTAACGTTATAATAG
HCOc102A [Int]      GTGAGTATAAAAACATGTCATTCTGGCATTTTAGGCCTTG--TTTCTAGAAAACCCCTCATATTTCTACAATATATGAATTAGCTAACGTTATGATAG
HCOc178A [Int]      GTGAGTATAAAAACATGTCATTCTGGCATTTTAAACATTGA-TTTCTAGAAAACCCCCATATTTCTACCGTATATGAATTAGCTGACGTTGTGATAG
HCOc061A [Int]      GTGAGCACAAAAACATGTCGTTCTGACATTTTATACCTTAA-TTTCTACAAACCCCCCTATATTTTTACAACATATAAATTAGCTAACGTTATGATAG
HCOc070A [Int]      GTAAGTATAAAAATATGTCGTTCCAGCATTTTAGACCTTGA-TTTCTAGAAAACCCCC--TATTTCTACGATATATGAATTAGCTAACATTATGATAG
HCOc378A [Int]      GTGAGTACAAAAGCATGTCATTCTGGCATTTTAGACCTTGA-TCTCTAAGAAAACCACTATATTTCTATAATATATTAATTGGCTAACGTTATGACAG
HCOc066A [Int]      GTGAGTATAAAAACATGTCGTTCTGGCATTTTAAACCTAAATTTTCTAGAAAATCTCCCATGTTTTTACAATATATGAATTAGCTAACATTATAACAG
HCOc224-c179 [Int]  GTGAGTATAAAAACATGTCGTTCTGGCATTTTAGACCTTGA-TTTCTAGAAGACCCCCATATTTCTACAAGATATGAATTAGCTAACGTTATGGTAG
HCOc047B (1)        GTGAGTATAAAAACATGTCGTTCTGGCATTTTCGACCTCAA-TTTCTAGAAAAATCGCCGTATTTCTATTAAATATGAATTAACTAACGTTATAACAG
HCOc052A [Int]      GTGAGTATAAAAACATATCGTTCTGGCATTTTAGACCTTTGATTTCTAGAAGACTCCCCATATTTCCAGTGTATATGAATTAGCTAACGTTATGACAG
HCOc058A [Int]      GTATGTATGAAGACATACCGTCTTGGCGTATTAGACCTTGA-TTTCTAGAAAGACCCCTGAGTTTTCACAATATAGGGATTAGCTAACGTTAGGACAG
HCOc236A [Int]      GTGAGTATACGAATATGTCGTTTTGTCACTTTAGACCTTGA-TTTCTAGAAAGATCCTCAAATGTCTTCAATGTAGGAATTAGCTAACGTCGTGACAG
HCOc271A [Int]      GTGAGTATAAAACTATGTCGTTTTGGCGTTTTAGACATTTGGTTTCTGGAAAGACCATCAAATCTC---AATATAAAATCAGCTAACGTTATGACAG
HCOc229B (1)        GTATGTATGAAAACGTGACATTTCGACATTT-AGA--TTTG-AATCTGAAAAGATCCTTAAAGTTCTATTATACCAGACCTAGCTAACGTTATGACAG
HCOc164A [Int]      GTATGTATAGATATATGGTGTGGTGGATTCCGGCCTTGC-TTCCTAGAAAAGCCTTCATTTCTCTATGATGCAGGAACCAGCTAATTTTATGACAG

consensus           GtgaGtAtaaaaacatgtcgTtctggcattttagaccTtra-tttCTagaaarnycccyawattTctayaatatadgaattagCTaAcgTtatgaYAG
```

Supplementary figure S1. Alignment of type-1 cropped sister introns with the internal introns of sister stwintrons. The type-1 cropped sister introns in *Hypoxylon* sp. CO27-5, HCOc047B (1) and HCOc229B (1), are shown in red. The sequences were aligned by MAFFT using the G-INS-i iterative refinement module with the PAM20 scoring matrix. Aligned positions that are only occupied in one or two of the 17 aligned U2 introns were removed and the manually trimmed alignment (98 informative nt) was shaded. 5'-Donor-, (predicted) BP- and 3' acceptor sequences are highlighted in magenta. The centrally located 10-nt palindrome is highlighted in yellow in the consensus line.

```
CO27-5 (HCOc066A; 207 nt)

GTGAggtgagtataaaaacatgtcgttctggcatttaaacctaaattttctagaaaatctcccatgtttttacaatatatgaattagctaacattataacagtaagttaagcgttcttatttcgtcgctatcgggattaagaaggcagtagctggtatattctagaaacgaaggatgaaaatgctgacacgacatgttctaatacattagCCTGC
|||| ||||||||||||||| |||||||||||||||| |||| || ||||||||||                                                                                                                |||| ||| ||||||||||||||||||||| ||||||| ||| ||||
GTGATgtgagtataaaaacacgtcgttctggcatttagaccttaa-tttctagaaa----------------------------------------------------------------------------------------------------------------cgaaagataaaaatgctgacacgacatgtttaatacactag-CTGC

EC38 (HECc034A; 93 nt)
```

Supplementary Figure S2. Alignment of sister stwintron HCOc066A (CO27-5) and the corresponding type-2 cropped sister intron HECc034A (EC38). The alignment illustrates the deletion associated with the derivation of a type-2 cropped sister intron in strain EC38 from a sister stwintron ancestor (similar to the one in CO27-5) at the same intron position in a gene encoding a putative integral membrane protein of 413 amino acids. The last uninterrupted codon of the upstream exon (Val) and the first uninterrupted codon of the downstream exon (Cys) are underlined. 5'-Donor-, (predicted) BP- and 3' acceptor sequences are highlighted in magenta or turquoise, respectively. The 10-nt palindrome is highlighted in yellow; The 5' copy in the stwintron and the single copy in the type-2 cropped sister intron are both perfect (i.e., 5'-TTTCTAGAAA). Note the change in intron phase from phase one (stwintron) to phase two (canonical intron).

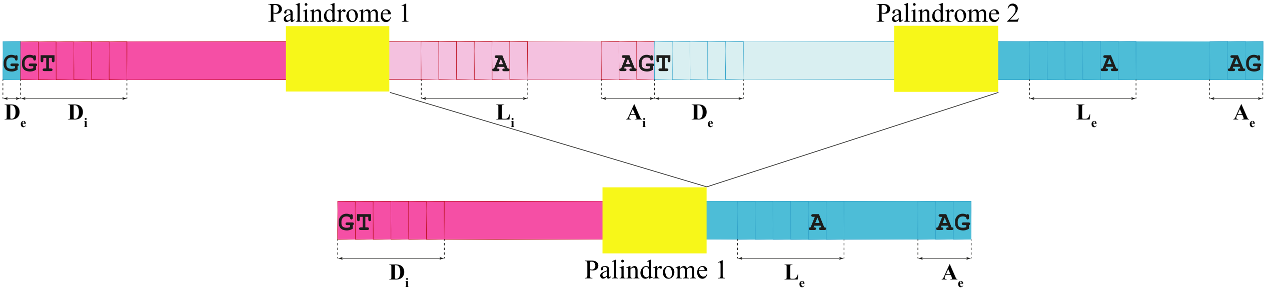

Supplementary Figure S3. Generation of a type-2 cropped sister intron from a [D1,2] sister stwintron during double-stranded DNA break (DSB) repair by microhomology-mediated end-joining (MMEJ). A double-stranded break occurs between the two copies of the 10-nt palindrome (yellow box) in the parent sister stwintron, where the crucial microhomology is (part of) that palindrome. Since the retained palindrome is perfect (5' W=T) we presume the imperfect 3' copy from the parent stwintron to be deleted.

```
EC38 HECc217A (Extra)     ggtgagtataaagacatgtcgttctggcattttagatcttgatttctgcaaaatcccccatgtt---------------tctacaatatatgaaccagctaacgttat-aacagtaagttaagcgttcttatttcgtcgctatcggggttaagaaggcagtacctggtatattctagaaacgaaggataaaaatgctgacacgacatgttttaatacatcag   206

EC38 HECc217b (Ortho)     ggtgagtatcaaaacatgccgtcctggcatttagaccttaatttctaagaactcctcccctttttccctccctaaacctctacaacataggaattaactaacgtcatgaacagtaagttaaccgttcatatctcgttggcatcggaattgagagggcaatgtctggtatatttcagaaaccaaggataaagatgctgacacgacatatc--aatacactag   220

CO27-5 HCOc004A (Ortho)    ggtgagtatcaaaacacgtcgtcctggcatttagtccttaatttctaaagaaaa----------aaaaaccctaaatttctacaacataggaataaactaacattatgaatagtaagttaaccgttcctatttcgttggcatcggggttaagaga--aatgtctagtacattccagaaaccaggataaaaatgctgacacgacatgtcttaatacactag   210
```

Sup**plementary Figure S4. Alignment of three sister stwintrons in a multicopper oxidase gene in *Hypoxylon* sp. CO27-5 and EC38.** Strain EC38 harbours a sister stwintron absent in CO27-5. The additional sister stwintron (HECc217A: phase two) is located 71 nt upstream of the sister stwintron (HECc217B: phase one), which corresponds to CO27-5 sister stwintron HCOc004A (~ 84.4 % identity). Identities between the top and the bottom sequences but not occurring in the EC38 3' stwintron, conserved in both fungi (middle sequence), are indicated with the colon signs. 5'-Donor-, (predicted) BP- and 3' acceptor sequences are highlighted in magenta or turquoise, and the 10-nt palindromes in yellow. The paralogue sister stwintrons in the EC38 gene are ~ 85.0 % identical.

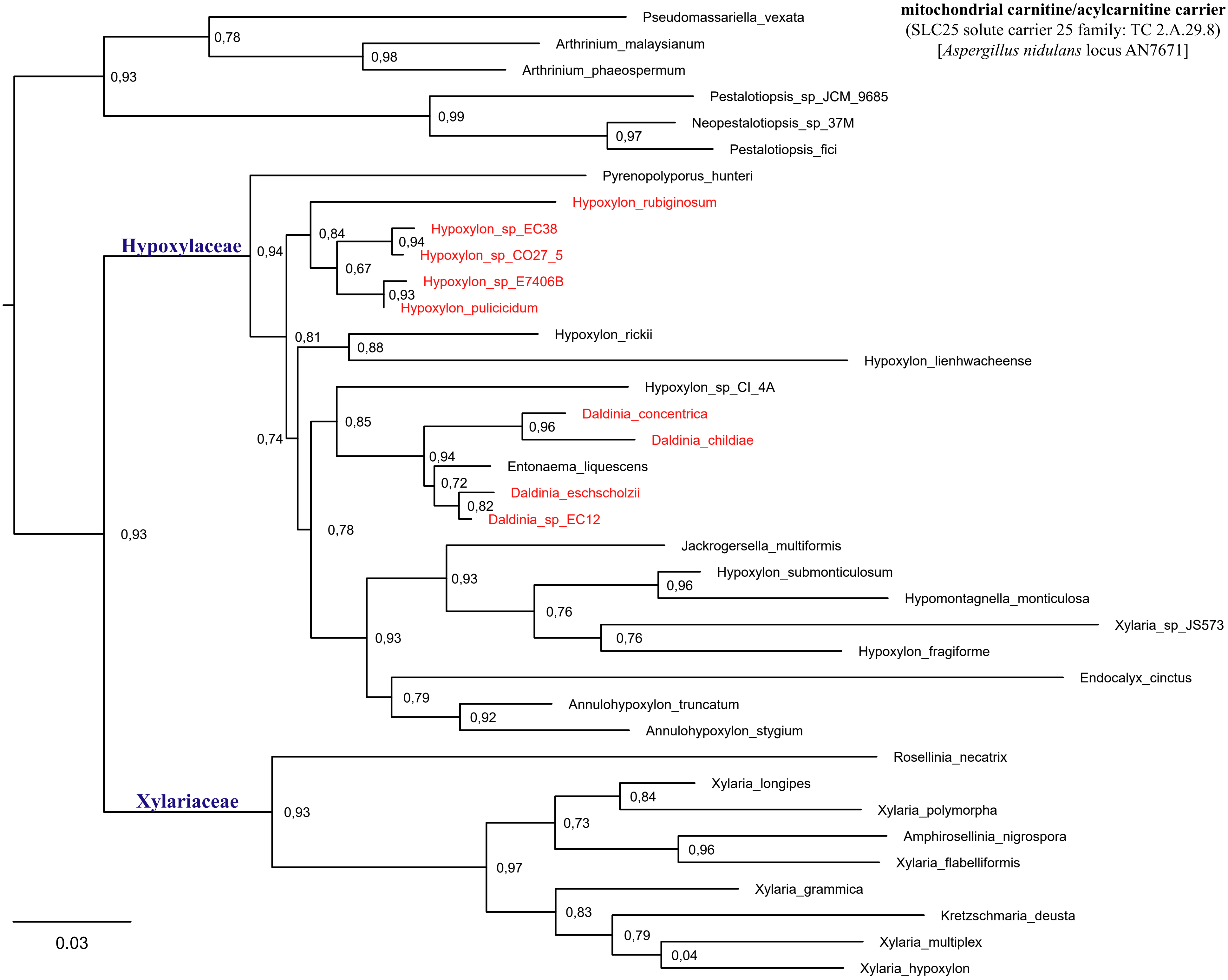

Supplementary Figure S5. Presence and absence of CO27-5-like sister stwintrons of high sequence similarity among Hypoxylaceae species. The names of species with recognisable sister stwintrons are printed in red lettering in taxon annotation of a maximum likelihood tree inferred for the well-conserved mitochondrial carrier protein (329 amino acids in *Hypoxylon* sp. CO27-5; cDNA: GenBank MW498246), encoded by the gene that carries sister stwintron HCOc017A. NCBI's WGS database was TBLASTN screened for the orthologue genes in another 36 Xylariales. Intron-exon structures were deduced guided by comparison of coding information. The orthologue proteins were aligned with MAFFT using G-INS-i iterative refinement and the BLOSUM 62 scoring matrix. This alignment was trimmed to 316 informative residues with Block Mapping and Gathering using Entropy (BMGE), using the BLOSUM 62 similarity matrix and block size 4. A maximum likelihood tree was then inferred with SMS-PhyML employing the LG + G + I + F substitution model. Branch support was estimated by Shimodaira-Hasegawa-like approximate likelihood-ratio tests (SH-like aLRT) (Anisimova and Gascuel 2006). The tree was rooted on the branch with the six species not assigned to the Hypoxylaceae or the Xylariaceae. The scale bar corresponds to an evolutionary distance of 0.03 substitutions per residue. The tree should not be considered authoritative molecular taxonomy for Hypoxylaceae.

Reference (aLRT node statistics):

Anisimova, M.; Gascuel, O. Approximate likelihood-ratio test for branches: A fast, accurate, and powerful alternative. *Syst. Biol.* **2006**, 55, 539–552.

***Hypoxylon* sp. CO27-5 cropped sister intron HCOc096A**

base pairing without GU = ~ 70.5 %; with GU = ~ 79.5 %

```
>RNA
-GUGAGUAUAAAAACAUGUCGUUCUAGCAUUUUAGACCUUAAUUUCUAGAAACGAAAGAUAAAAAUGCUGACAUGAUAUGUUUUAAUACACUAG
GAUCACAUAAUUUUGUAUAGUACAGUCGUAAAAAUAGAAAGCAAAGAUCUUUAAUUCCAGAUUUUACGAUCUUGCUGUACAAAAAUAUGAGUG-
REV<
```

***Hypoxylon* sp. CO27-5 sister stwintron HCOc066A**

base paring in the TIRs (only):
without GU ~ 72.2 %; with GU = ~ 77.8 %

```
>RNA
GUGA-GUAUAAAAACAUGUCGUUCUGGCAUUUUAAACCUAAAUUUUCUAGAAAAUCUCC-CAUGUUUUUACAAUAUAUGAAUUAGCUAACAUUAUAACAGUAAGUUAAGCGUUCUUAUUUCGUCGCUAUCGGGAUUAAGAAGGCAGUAGCUGGUAUAUUCUAGAAACGA-AGGAUGAAAAUGCUGACACGACAUGUUCUAAUACAUUAG
GAUUACAUAAUCUUGUACAGCACAGUCGUAAAAGUAGGAA-GCAAAGAUCUUAUAUGGUCGAUGACGGAAGAAUUAGGGCUAUCGCUGCUUUAUUCUUGCGAAUUGAAUGACAAUAUUACAAUCGAUUAAGUAUAUAACAUUUUUGUACCCUC-UAAAAGAUCUUUUAAAUCCAAAUUUUACGGUCUUGCUGUACAAAAAUAUG-AGUG
REV<
```

***Daldinia childiae* sister stwintron Dchc003A**

base paring in the TIRs (only):
without GU = ~ 68.7 %; with GU = ~ 79.2 %

```
>RNA
-GUAUGUAUGAAAACAUCCCGUGUUAUUCUUUUGAAACCCCCUUUCUAGAACGGUUUCGGAACCACCUAGGUAUACAGUAGCUAACGCUCAGUAGUAAGUGUGAUGGCCCUAUCAUUUCGACUAAAUAAAAAAAGAACAGUGAAGAUUCUAGAAUUGUAGUAAUGCAAAGCUGAUACGAGAUGUUUUCAUACAUUAG
GAUUACAUACUUUUGUAGAGCAUAGUCGAAACGUAAUGAUGUUAAGAUCUUAGAAGUGACAAGAAAAAAAUAAAUCAGCUUUACUAUCCCGGUAGUGUGAAUGAUGACUCGCAAUCGAUGACAUAUGGAUCCACCAAGGCUUUGGCAAGAUCUUUCCCCCAAAGUUUUCUUAUUGUGCCCUACAAAAGUAUGUAUG-
REV<
```

Supplementary Figure S6. Base pairing in double-stranded RNA of two linear molecules of the same single-stranded sister (stw)intron RNA. The three example sister (stw)introns are the same as those in Figure 4a.Watson-Crick base pairing is indicated with the colon sign. Non-canonical GU base pairing is highlighted in red. The terminal inverted repeats (TIRs) are indicated by horizontal arrows. Percentages of base paired nucleotides are given with and without GU pairing (TIRs only).

Daldinia childiae Dchc003A (sister stwintron)

```
ggtatgtatgaaaacatcccgtgttattcttttgaaacccccttttctagaacggtttcggaaccacct-----aggtatacagtagctaacgctcagtagtaagtgtgatggccctatcatttcgactaaataaaaaaagaacagtgaagattctagaattgtagtaatgcaaagctgatacgagatgttttcatacat-tag 197 nt
|||||||||||||  |||| ||||| || ||||  | |||| |||| |   | || ||| ||| ||||     ||||||| ||| ||||    |||||||    ||||||||||||||||||||||| ||||  |||||  |  ||| | |||||| ||||||| || ||||||||||| || | |||||||| |||||  ||
Ggtatgtatgaaaggatcctgtgttgttattttcgagcccctttttccatggcagtctcgaaactaccttcgtaaggtatatagtggcta----tcagtag----tgtgatggccctatcatttcgacgaaatcgaaaaattatggtggaaattctaaaattgtaatattgcaaagctgacactaaatgttttcgtacatgcag 195 nt
```

Daldinia childiae Dchc003S (long sister intron)

Supplementary Figure S7. Alignment of the *Daldinia childiae* sister stwintron Dchc003A and the long sister intron Dchc003S. The alignment shows the divergence of a long canonical sister intron from a sister stwintron of very similar length. Both intervening sequences are in sequence contig VYXO01000003 (see Supplementary Table S1, sheet Daldinia). Sister stwintron Dchc003A is at the seventh intron position in the gene for a GTP-binding protein of the dynamin family. The canonical long sister intron Dchc003S is at the first intron position in the gene for an arylsulfatase. Donor-, BP- and 3' acceptor sequences are colour-coded. The internal splice sites of the stwintron (top) are absent from the canonical intron (bottom), seemingly the result of two small deletions. Note that the 5' G in the lower sequence (capital letter) is exonic.

Supplementary Table S2. Oligonucleotide primers used in this study.
Oligonucleotide primers for cDNA and/or gDNA sequencing.

Gene Hypoxylon sp. CO27-5	Name of oligo	Sequence (5'-)
c047B (I)	c047B_seq_F	ACCGGCGCTAATTTCCTAAT
c047B (I)	c047B_seq_R	GCATCGTATCCCACGCTATT
c153A (II)	c153A_seq_F	GCTAGCAACAGAAAAGCAAC
c153A (II)	c153A_seq_R	AGTTGCATGTGTGGTTATGA
c229A (I)	c229A_seq_F	TGACCGAACAACAGCAACAG
c229A (I)	c229A_seq_R	GTCTATCGGCATACCTCGCT
c105A (II)	c105A_seq_F	TTACTAGCCGGCGGATAAGG
c105A (II)	c105A_seq_R	ATTACATTGCCCCGGATCCA
c164B (II)	c164B_seq_F	TATGCCCAGAGAGCCATTGT
c164B (II)	c164B_seq_R	TCACCCGAATTGTCCTCTTGA
c343A (II)	c343A_seq_F	AGAGGGGCAAACGTCATCTA
c343A (II)	c343A_seq_R	CGCTGAGTACATGCCTACCT
c024A (II)	c024A_seq_F	TCGAGAGTCGCGCCGGAATC
c024A (II)	c024A_seq_R	TAAGTCTGCCAGGAGATCGC
c147A (II)	c147A_seq_F	ATCCTCAAGCCGCCTCATAA
c147A (II)	c147A_seq_R	GCATCACAACATAATTGCACGA
c121A (II)	c121A_seq_F	AAGGAGAAGGAACCAGAGCC
c121A (II)	c121A_seq_R	CGCAACGCCTCTCATCTAAC
c004A	c004A_seq_F	CATCGTTCCTATGTAATTAT
c004A	c004A_seq_R	AGAGCTGTAATAGCTATCCT
c017A	c017A_seq_F	GTACCCCAATGTAGTATACG
c017A	c017A_seq_R	CAGATCGCATAACTCGATGA
c017B	c017B_seq_F	GAACAGTGTCTTCGTCAATCT
c017B	c017B_seq_R	GTTACCGCAAGATTGTTAGAC
c021A	c021A_seq_F	ATGATACAGAATCTCTAGTCG
c021A	c021A_seq_R	ACATGTTGCTTCCACTGTTTC
c052A	c052A_seq_F	CATTTCCTACCCTTGGAGAAG
c052A	c052A_seq_R	AGGCCATCATACTCATCCATC
c061A	c061A_seq_F	GACGTCGAGGTTTACCTAAC
c061A	c061A_seq_R	ATTTACGGTATATACCATTC
c066A	c066A_seq_F	CTTGCTAGAGACACTCTCTA
c066A	c066A_seq_R	TCCCTAGGTTCCAAGGAGAC
c070A	c070A_seq_F	GATGAGGTGGTGCAGTTGCG
c070A	c070A_seq_R	TCAGAAACTCATCGACTACAG
c076A	c076A_seq_F	CGACGTCAGCATCCCTACTA
c076A	c076A_seq_R	AGGTTTCCCACGTCAAACAG
c102A	c102A_seq_F	CCTAGGTATTTACACACCATG
c102A	c102A_seq_R	TCCCTCTCATGTAGCTCAAG
c164A	c164A_seq_F	CAATTCTTTACGATATAGATA
c164A	c164A_seq_R	CATTATTTAGACATTGCAGCAG
c178A	c178A_seq_F	ACAGTCAACTTATAAAAGCTC
c178A	c178A_seq_R	CTAACTGTAACTCCTATCGG
c236A	c236A_seq_F	GCTCTCCACAGTCGAGTCTCA
c236A	c236A_seq_R	CTAGCGACGTAATATAACCGA
duf636	duf636_seq_F	GACTTACTACTCGGAACTCC

duf636	duf636_seq_R	GTACTTGGATAAAACCTTT
c252A	c252A_seq_F	CAGGTCAAGAGGCTTCATCAC
c252A	c252A_seq_R	TGCTCCAGCGTATAATAGATG
c271A	c271A_seq_F	GCTCTGCGTATGTTGTGGAA
c271A	c271A_seq_R	AGAGAATGGGGAGAAGCACA
c332A	c332A_seq_F	GCATATTTTGCGCCACTTCT
c332A	c332A_seq_R	TTCTAACACAGCGGGAGTCA
c378A	c378A_seq_F	AGGTTTGCTGTAGATGAGAG
c378A	c378A_seq_R	AAGTACCACACAGAGATATT
c406A	c406A_seq_F	GAGATTGCACCGGCTCTCAC
c406A	c406A_seq_R	CTTGCCGCAAAACTACCAAC
c522A	c522A_seq_F	GAAGACGTTCCCTAGTGCTA
c522A	c522A_seq_R	GACATATCTCTCAGGACGGA
c004B	c004B_seq_F	AAGGACTGTCGCCCATGAT
c004B	c004B_seq_R	CAAGAACTCCTAGGCCATCG
c016A	c016A_seq_F	CATCGGATAAAGCTCGAGGCCT
c016A	c016A_seq_R	CACGGGCAGCACCCAATTTAAC
c016B	c016B_seq_F	ACTTGCTATGTCCAAGCTTGT
c016B	c016B_seq_R	TACATTCTAGATTACGTATC
c020A	c020A_seq_F	TCCAACTCCACCTCGTCTTC
c020A	c020A_seq_R	AATGTTGGTAGCGATGAGGC
c024B	c024B_seq_F	TCATTGATCTTCGCTCCGTA
c024B	c024B_seq_R	TAGATGTATCTCTAGATCTA
c046A	c046A_seq_F	CAGAAGCATTCCAAAAAGGAAC
c046A	c046A_seq_R	CTGGACAAAACACCATTCGTC
c091A	c091A_seq_F	GTAAACATCTTGTGTCAAAAC
c091A	c091A_seq_R	TAGGTACGTTAATTATATCAT
c103A	c103A_seq_F	GCATGACTGAAAGCCTCACA
c103A	c103A_seq_R	CGCAGGCAGAAAATGGACTA
c159A	c159A_seq_F	GTTCGTCTTTCGATAGTTCGT
c159A	c159A_seq_R	GTGCAAGGTGAACACTTGAAC
c263A	c263A_seq_F	CAACGGTTCTAGCCTGAGGA
c263A	c263A_seq_R	ATTCGCCCTTTTGAGATGAA
c304A	c304A_seq_F	GAGAACTTAAATAGTTCGATAT
c304A	c304A_seq_R	CGACGTATCTCTAGAATATTTC
helQ	helQ_seq_F	GCATCTTGCCAGCGTCTATC
helQ	helQ_seq_R	CACAAGGCAAGGTACAGAAG

Oligonucleotide primers for RT-PCR verification of stwintron splicing intermediates.

**Gene Hypoxylon sp. CO27-5**	**Name of oligo**	**Sequence (5'-)**
c004A	c004A_splinter_F	ATGGCCAAAACGAACAATCC
c004A	c004A_splinter_R	GTGTATTAAGACATGTCGTG
c017A	c017A_splinter_F	GACTGCAGATTCAGTACTCG
c017A	c017A_splinter_R	AGTATTAAAACATGTCGCGTC
c017B	c017B_splinter_F	GTACTTGGGGATATCAGGATC
c017B	c017B_splinter_R	TAAAACACGTCGTGTCAGTAC
c052A	c052A_splinter_F	CATTTCCTACCCTTGGAGAAG
c052A	c052A_splinter_R	TAAAACATGTCGTATTAGCAT

c061A	c061A_splinter_F	GACGTCGAGGTTTACCTAAC
c061A	c061A_splinter_R	GTATTAAAACATATCGTGTTA
c043A	c043A_splinter_F	GAAATATTGCGACAGACATC
c043A	c043A_splinter_R	GTATTAGAACATGTCGTGTC
c070A	c070A_splinter_F	CAATACCATTATGTGGCGATG
c070A	c070A_splinter_R	GTGTTAGCATTTTCATCCTTC
c076A	c076A_splinter_F	GCATGGTCGGATAATGAAAAA
c076A	c076A_splinter_R	CCTAATCCCGATAACGACGA
c102A	c102A_splinter_F	ATGGTATTTACTGTAATATAT
c102A	c102A_splinter_R	GTGTATTAAAACATGTCGTGTC
c164A	c164A_splinter_F	GTTCACTACATCGAAGACCAG
c164A	c164A_splinter_R	GTAGTGTCAATATCTATAGTC
c178A	c178A_splinter_F	AAAAACATGTCATTCTGGCATT
c178A	c178A_splinter_R	TCGTCCCAACCTAAACATCC
duf636	duf636_splinter_F	CCTAGGTAGCTATATGCTGT
duf636	duf636_splinter_R	GCATGTTGGACAGACTGATG
c236A	c236A_splinter_F	GCTCTCCACAGTCGAGTCTCA
c236A	c236A_splinter_R	TAACTTGTTAGCATTTTAACAC
c252A	c252A_splinter_F	CAGGTCAAGAGGCTTCATCAC
c252A	c252A_splinter_R	AGGATATTACAACATATCGTG
c271A	c271A_splinter_F	GCACTCGTACGGTCTTCTCC
c271A	c271A_splinter_R	GCGTCGTGTCAGCATTTTTA
c332A	c332A_splinter_F	GCATATTTTGCGCCACTTCT
c332A	c332A_splinter_R	CATTCTTGGCCTTTGCTTCT
c378A	c378A_splinter_F	AGGTTTGCTGTAGATGAGAG
c378A	c378A_splinter_R	ATTAAAACATGTCGTGTCAG
c406A	c406A_splinter_F	ATCCTCAATGCGACGAGCGG
c406A	c406A_splinter_R	GCATGTCGTGTCAGCATTGT
c522A	c522A_splinter_F	ATGAAGTGGTATACTCTACTT
c522A	c522A_splinter_R	TCCTGTCAGCAATTTTGACTTC
c004B	c004B_splinter_F	AAGGACTGTCGCCCATGAT
c004B	c004B_splinter_R	TGTATGAAACGCGTCATATCAGT
c016A	c016A_splinter_F	GAGACCTGCCGGAACGTTGT
c016A	c016A_splinter_R	GTTTACCTTGGACCGTTTGAG
c016B	c016B_splinter_F	GTCATTGATCTGACCTCCAT
c016B	c016B_splinter_R	TGTTAGCTCTCTCCAGCTGA
c020A	c020A_splinter_F	GATCCCATGACACGAACAGG
c020A	c020A_splinter_R	CCGTGTCAGTACTTCTAACTCC
c024B	c024B_splinter_F	GTCGCGATTAAGCTGCAGAAG
c024B	c024B_splinter_R	TCATTGAGCCCTTAATCCGTA
c046A	c046A_splinter_F	CAGAAGCATTCCAAAAAGGAAC
c046A	c046A_splinter_R	GGAAAACATCTTGTGTCAGC
c091A	c091A_splinter_F2	CGCCTCAGCGGAGCAGATCTA
c091A	c091A_splinter_R2	GTTCGTCAGTAATCTTATAAC
c091A	c091A_splinter_F1	ACGGCGAGCATTCCATCGTTC
c091A	c091A_splinter_R1	AGGTATGTATTAGTGTCGGTG
c103A	c103A_splinter_F	ATCCTGCGGATGCATCTAAC
c103A	c103A_splinter_R	TATACCAGGCGGTGCTTTCT

c159A	c159A_splinter_F	CGATACTAGGCTTCATAAATAC
c159A	c159A_splinter_R	ATACCAGAGTATATCATGTCA
c263A	c263A_splinter_F	TCGCTTTCCCGAGACTAAGA
c263A	c263A_splinter_R	TAGGGGTGACCATTCCACAT
c304A	c304A_splinter_F	GAGATACATTGTCCCACCAAC
c304A	c304A_splinter_R	CAGCAATCAGCCTAGCCATG
helQ	helQ_splinter_F	GCACATCTATAGCACATTCT
helQ	helQ_splinter_R	CTAAATGTTAGCTTACCTGA

Supplementary Table S3. List of RNA SRAs that illustrate alternative excision of sister stwintrons in one U2 reaction with the retention the 5'-G_1 of the external intron

**CO27-5 [D1,2] sister stwintron**	**SRA Alternative splicing event**
HCOc271A	SRR1801290.13818186
HCOc004A (alternative 3′ splice)	SRR1801288.17896446
HCOc017A (alternative 3′ splice)	SRR1801291.21659993
HCOc236A (alternative 3′ splice)	SRR1801290.14509435
HCOc378A (alternative 3′ splice)	SRR1801290.31839927
HCOc004B (alternative 3′ splice)	SRR1801290.29352535
**EC38 [D1,2] sister stwintron**	**SRA Alternative splicing event**
HECc145A	SRR1798129.1669524
HECc326A	SRR1798132.10935711
HECc010A (alternative 3′ splice)	SRR1798131.9126740
HECc011A (alternative 3′ splice)	SRR1798129.17227791
HECc140A (alternative 3′ splice)	SRR1798130.18795761
HECc217B (alternative 3′ splice)	SRR1798131.15498780
HECc296A (alternative 3′ splice)	SRR1798128.3418214

In all cases but HCOc004B, HECc296A and HECc140A the alternative splicing reaction leads to premature translation termination codons and a C-terminally truncated peptide product, ordinarily provoking nonsense-mediated mRNA decay of the product mRNA.
